# Supplementary figures and images for: Allosteric activation of the co-receptor BAK1 by the EFR receptor kinase initiates immune signaling
Source: eLife. 2024 Jul 19;12:RP92110. doi: 10.7554/eLife.92110 (PMC11259431; doi:10.7554/eLife.92110)

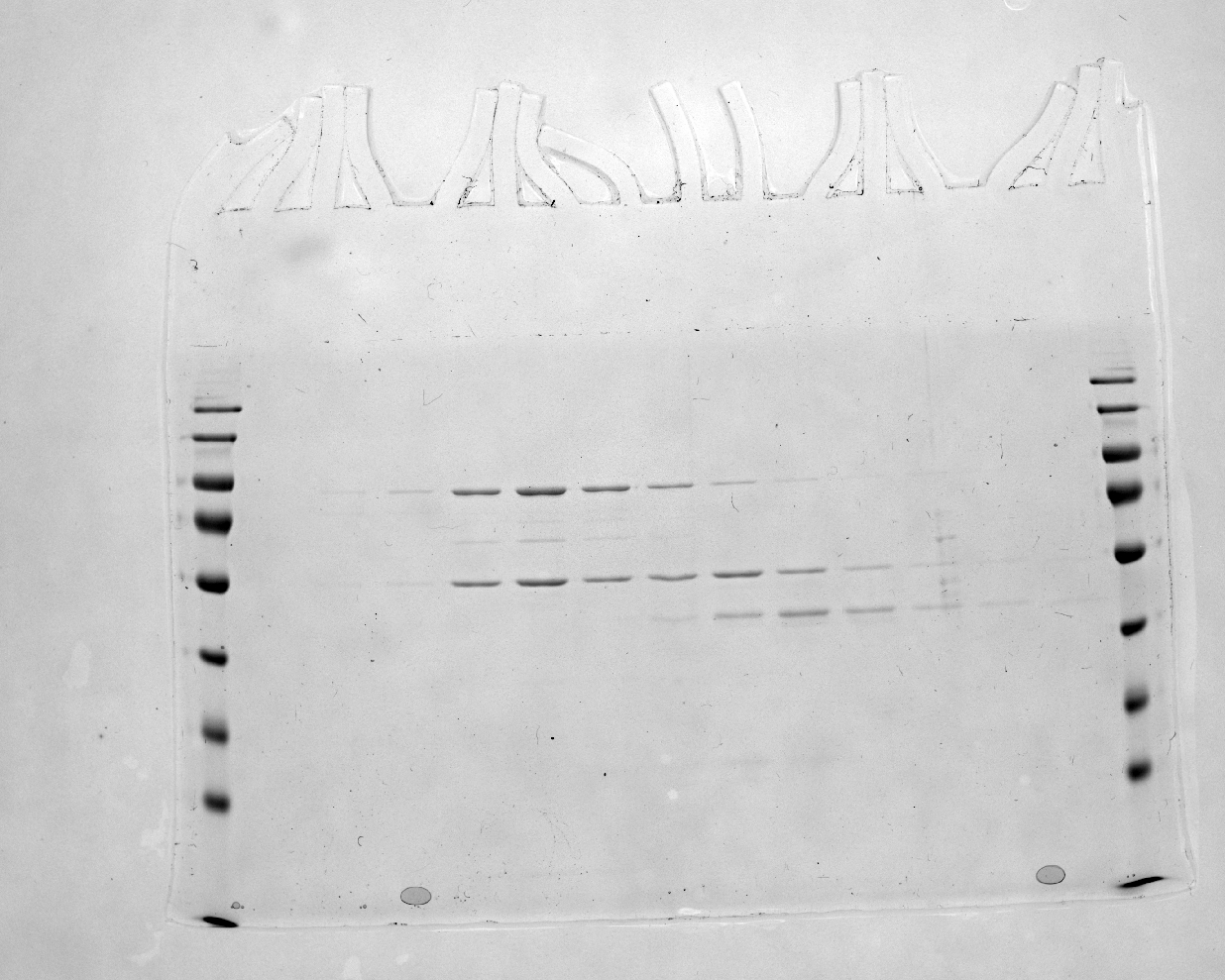

Supplement: Figure 1—source data 1. [file elife-92110-fig1-data1.zip › user 2023-07-30 15h13m01s(Coomassie Blue).jpg]

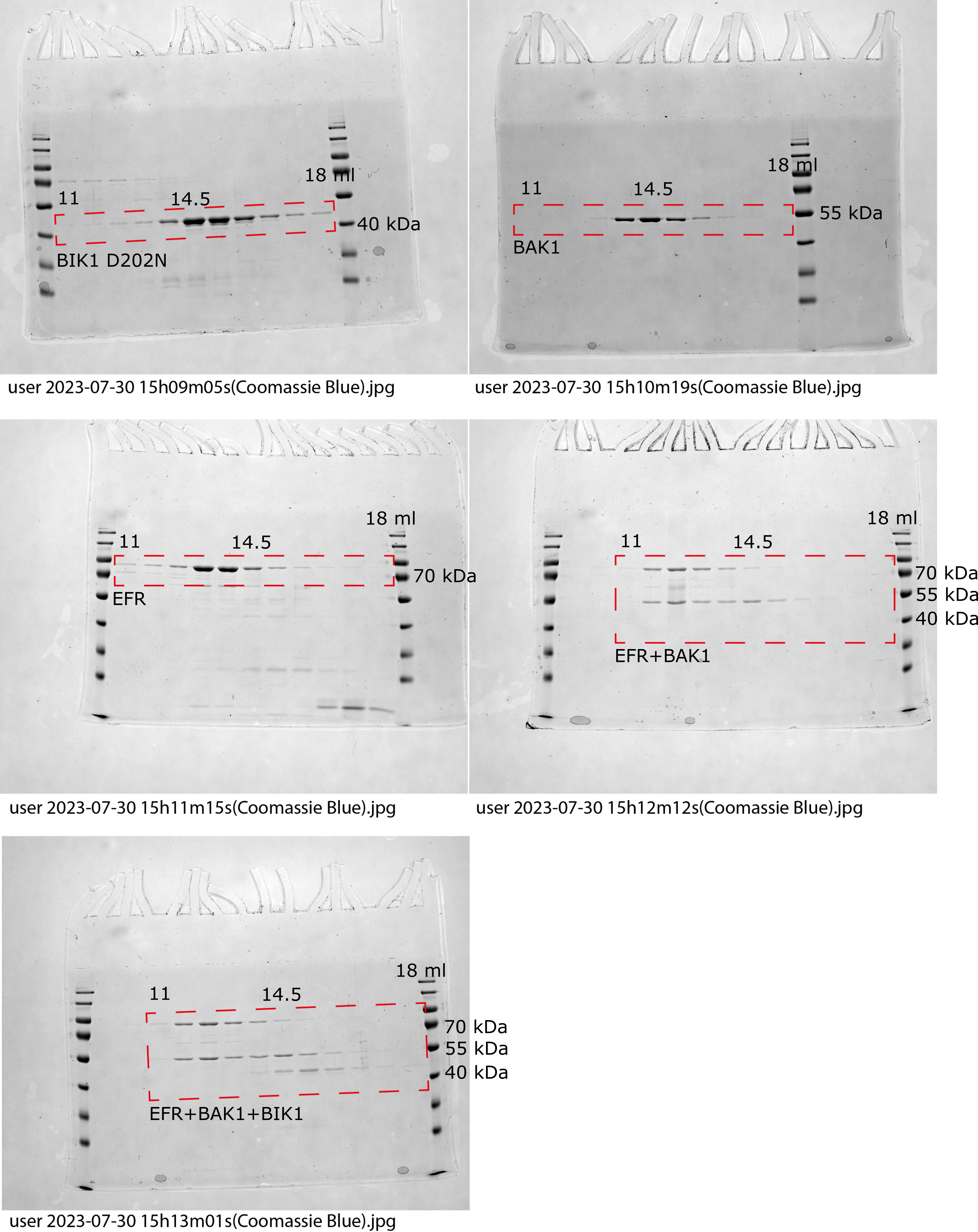

Supplement: Figure 1—source data 1. [file elife-92110-fig1-data1.zip › annotated.png]

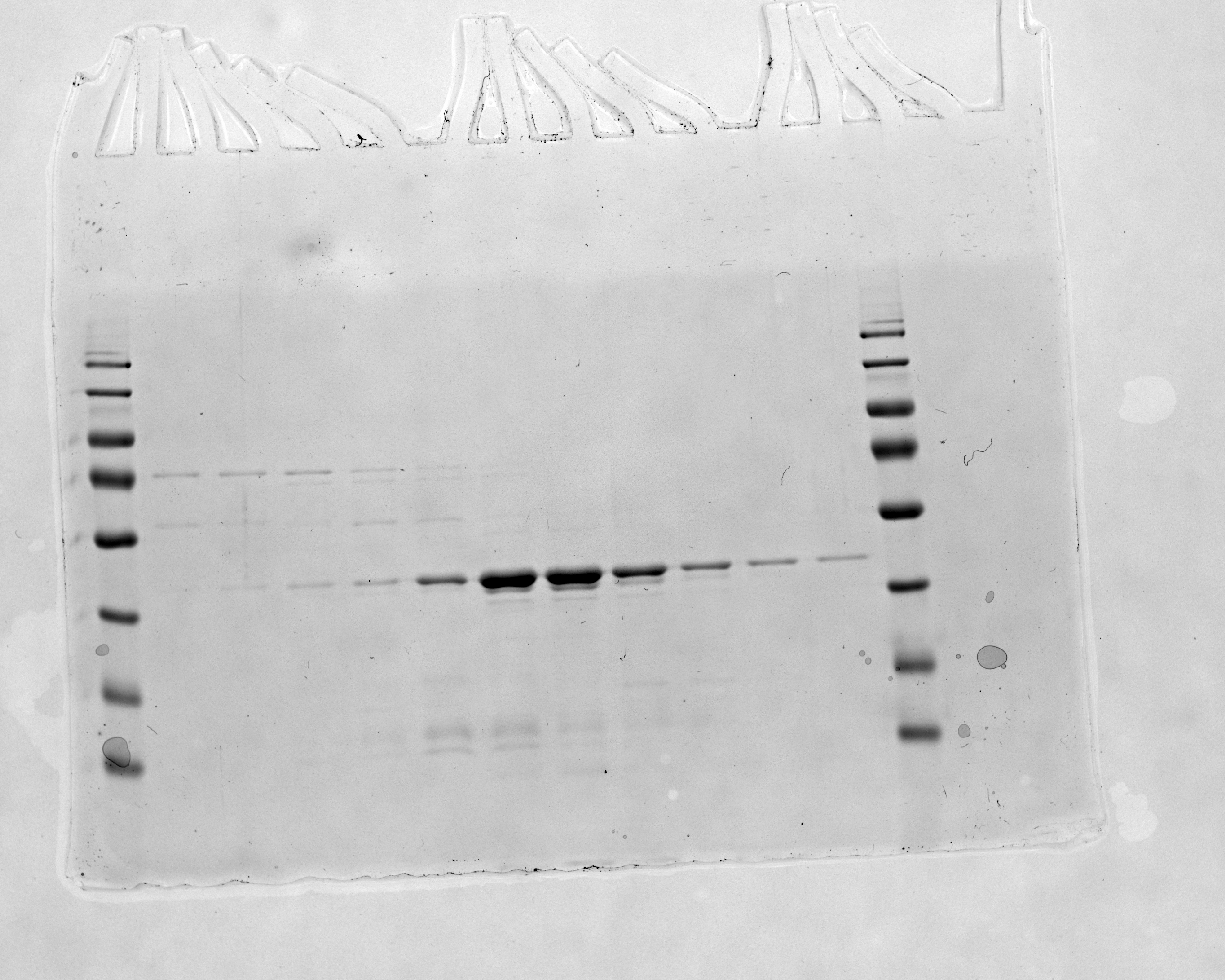

Supplement: Figure 1—source data 1. [file elife-92110-fig1-data1.zip › user 2023-07-30 15h09m05s(Coomassie Blue).jpg]

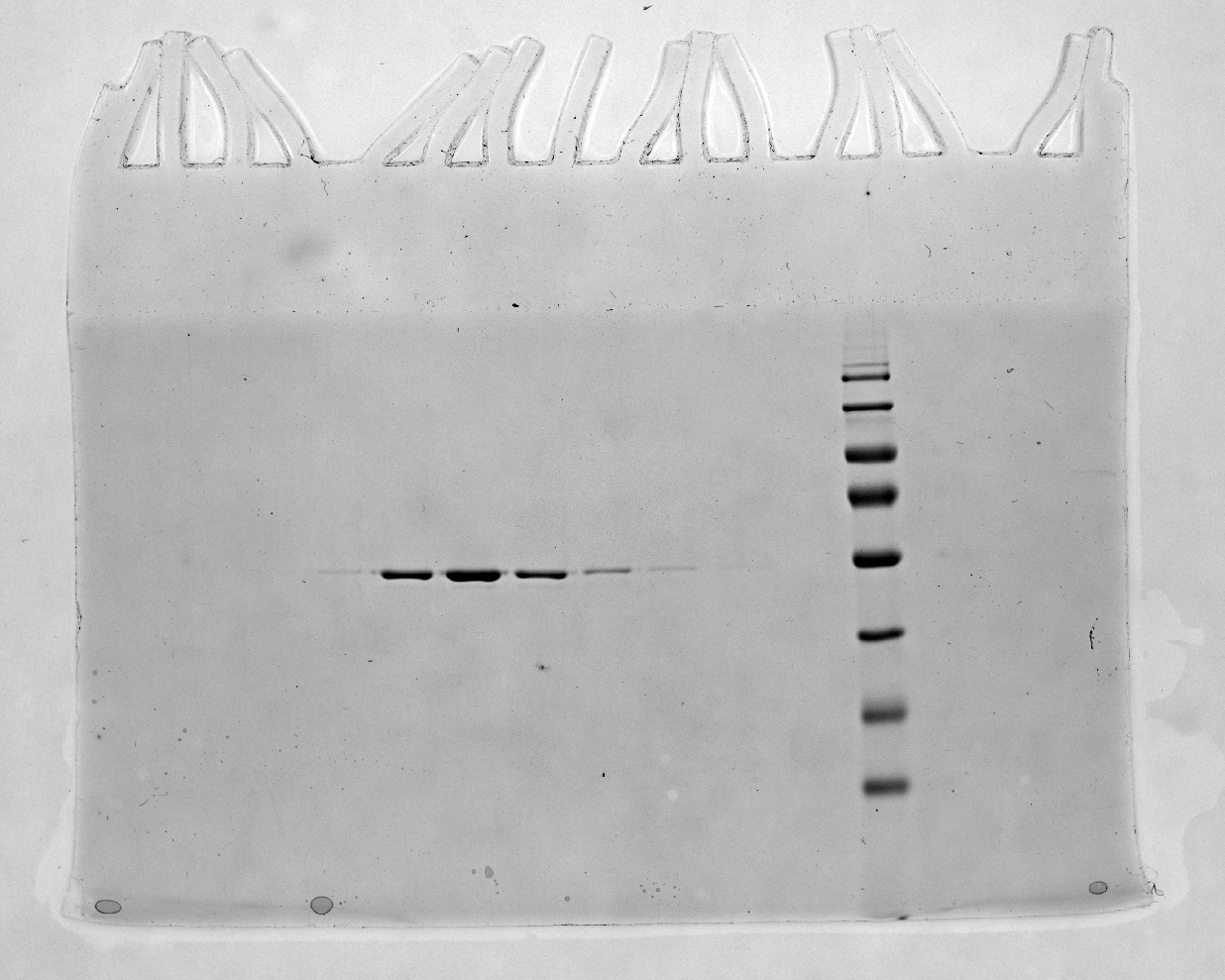

Supplement: Figure 1—source data 1. [file elife-92110-fig1-data1.zip › user 2023-07-30 15h10m19s(Coomassie Blue).jpg]

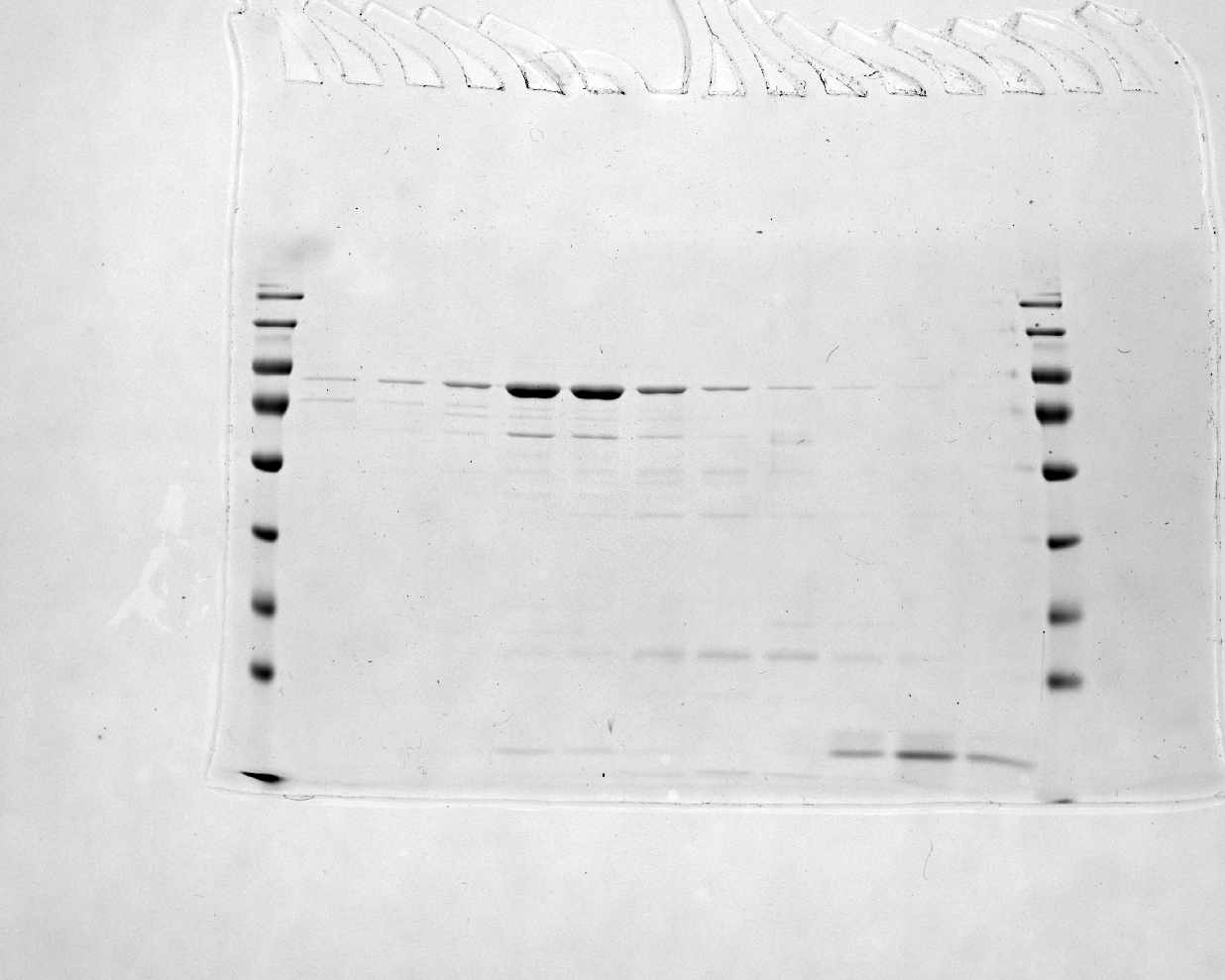

Supplement: Figure 1—source data 1. [file elife-92110-fig1-data1.zip › user 2023-07-30 15h11m15s(Coomassie Blue).jpg]

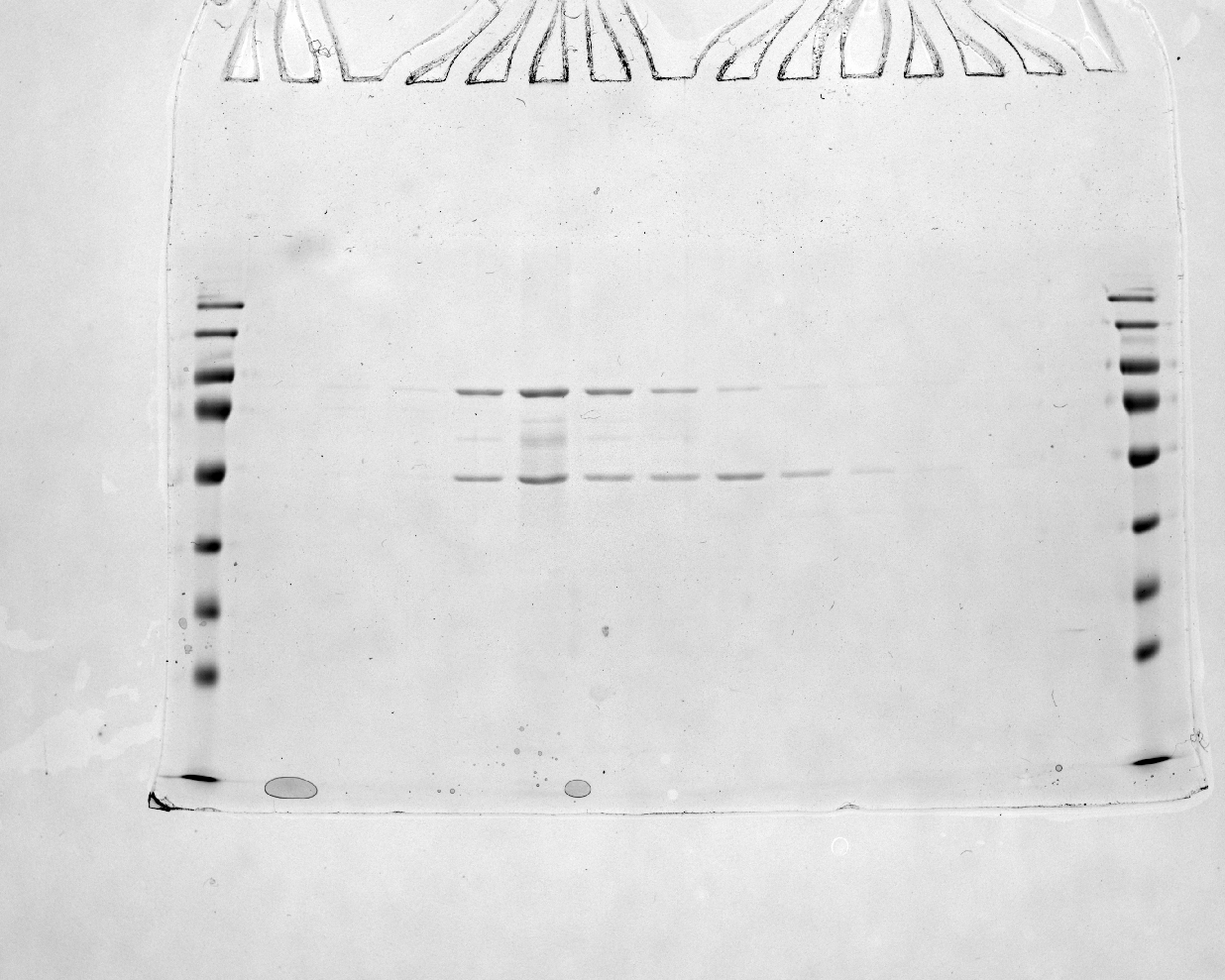

Supplement: Figure 1—source data 1. [file elife-92110-fig1-data1.zip › user 2023-07-30 15h12m12s(Coomassie Blue).jpg]

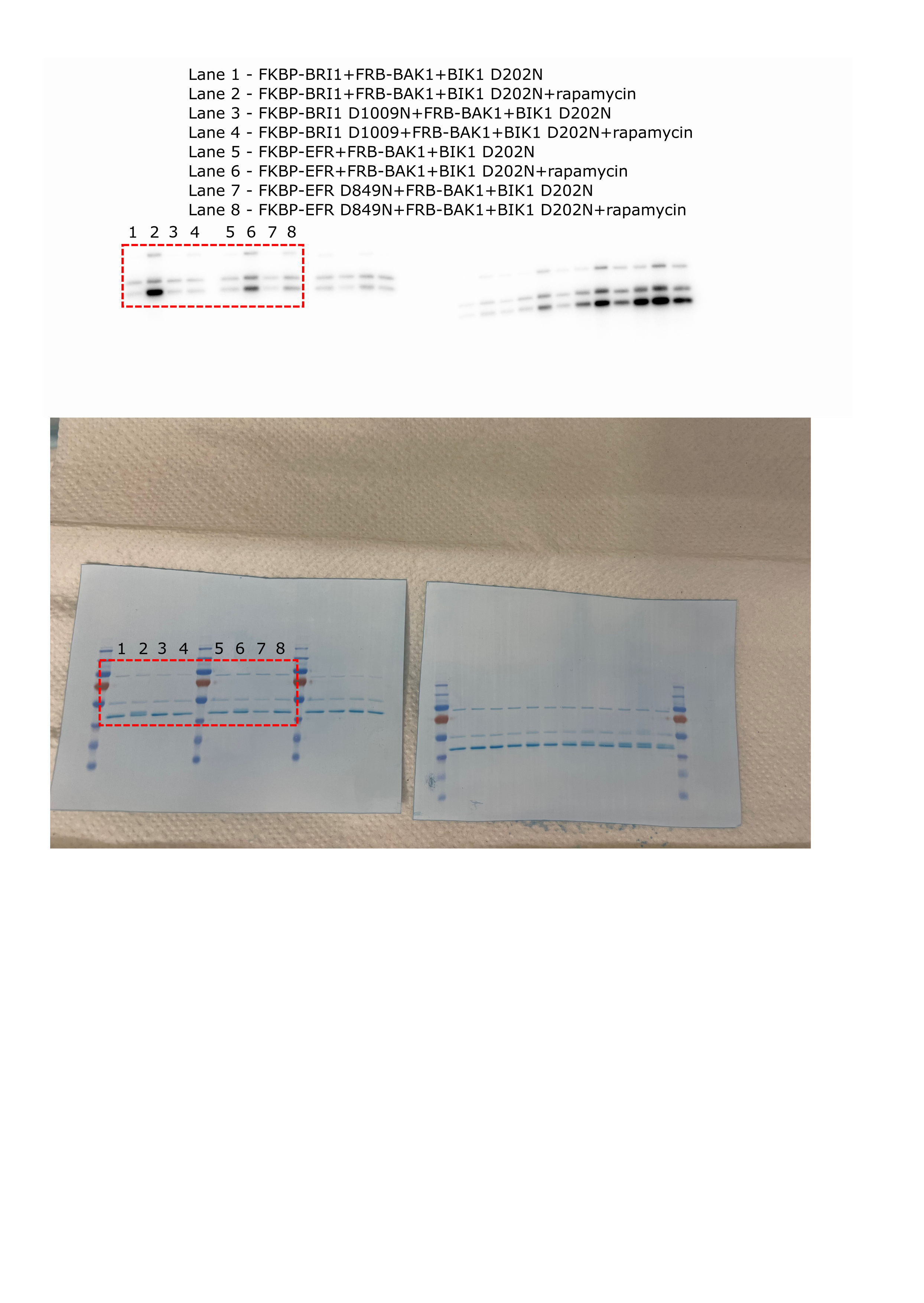

Supplement: Figure 1—source data 2. [file elife-92110-fig1-data2.zip › annotated.png]

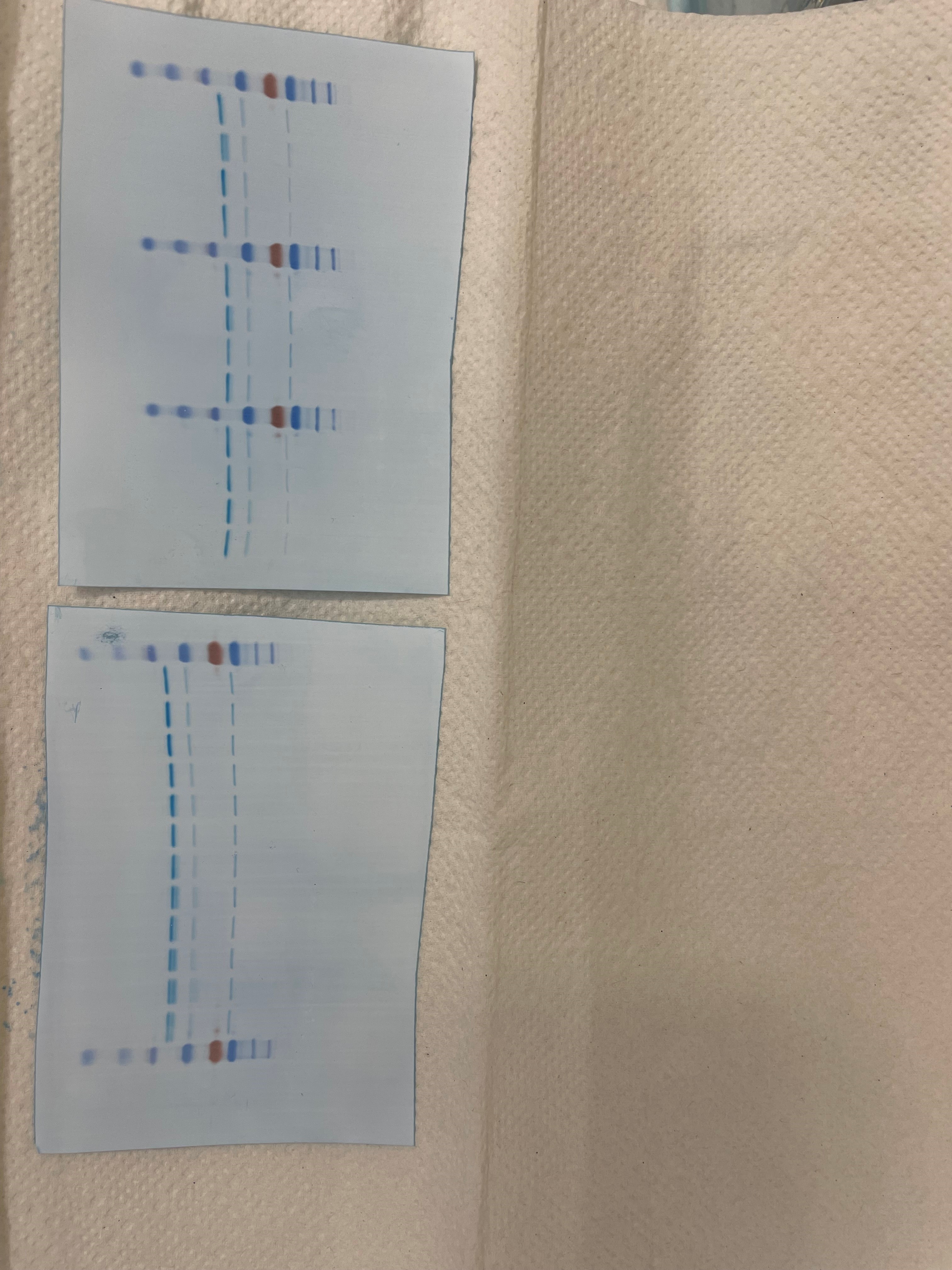

Supplement: Figure 1—source data 2. [file elife-92110-fig1-data2.zip › 2023-06-24_inVitroRiD_BRI1_FLS2_catalyticReqTest_CBBG.jpg]

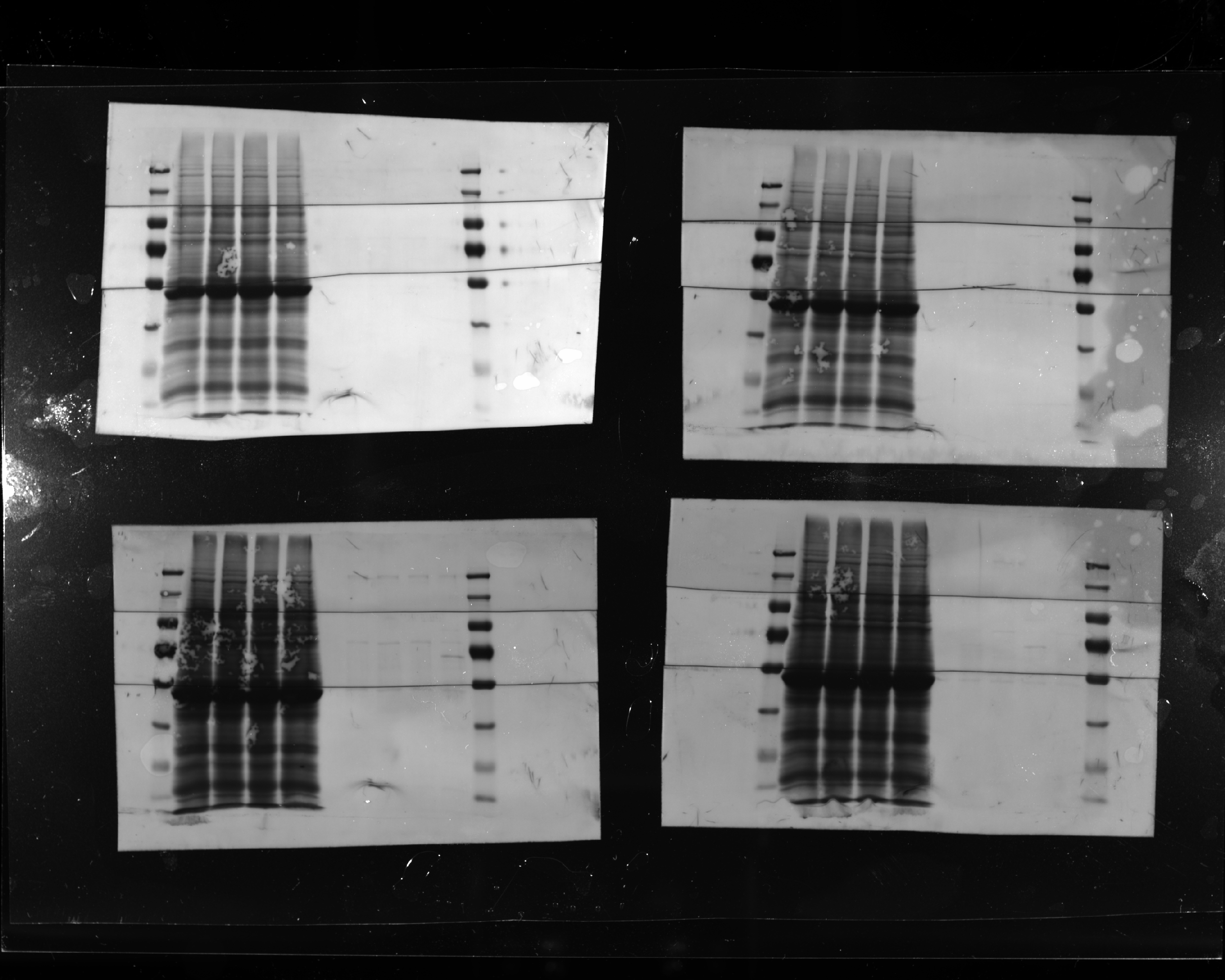

Supplement: Figure 1—figure supplement 1—source data 1. [file elife-92110-fig1-figsupp1-data1.zip › user 2021-05-26 13h53m13s(Colorimetric).jpg]

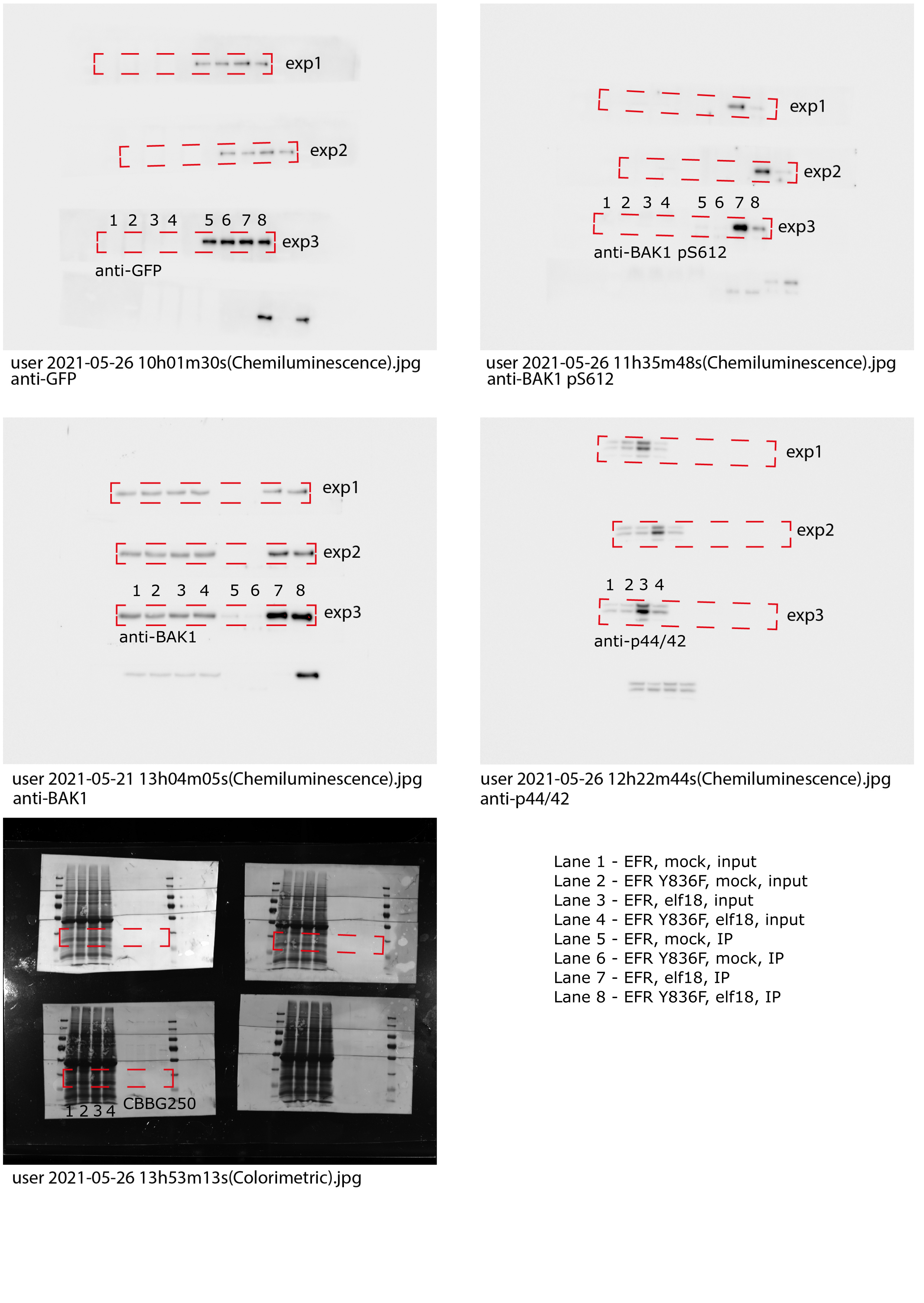

Supplement: Figure 1—figure supplement 1—source data 1. [file elife-92110-fig1-figsupp1-data1.zip › annotated.png]

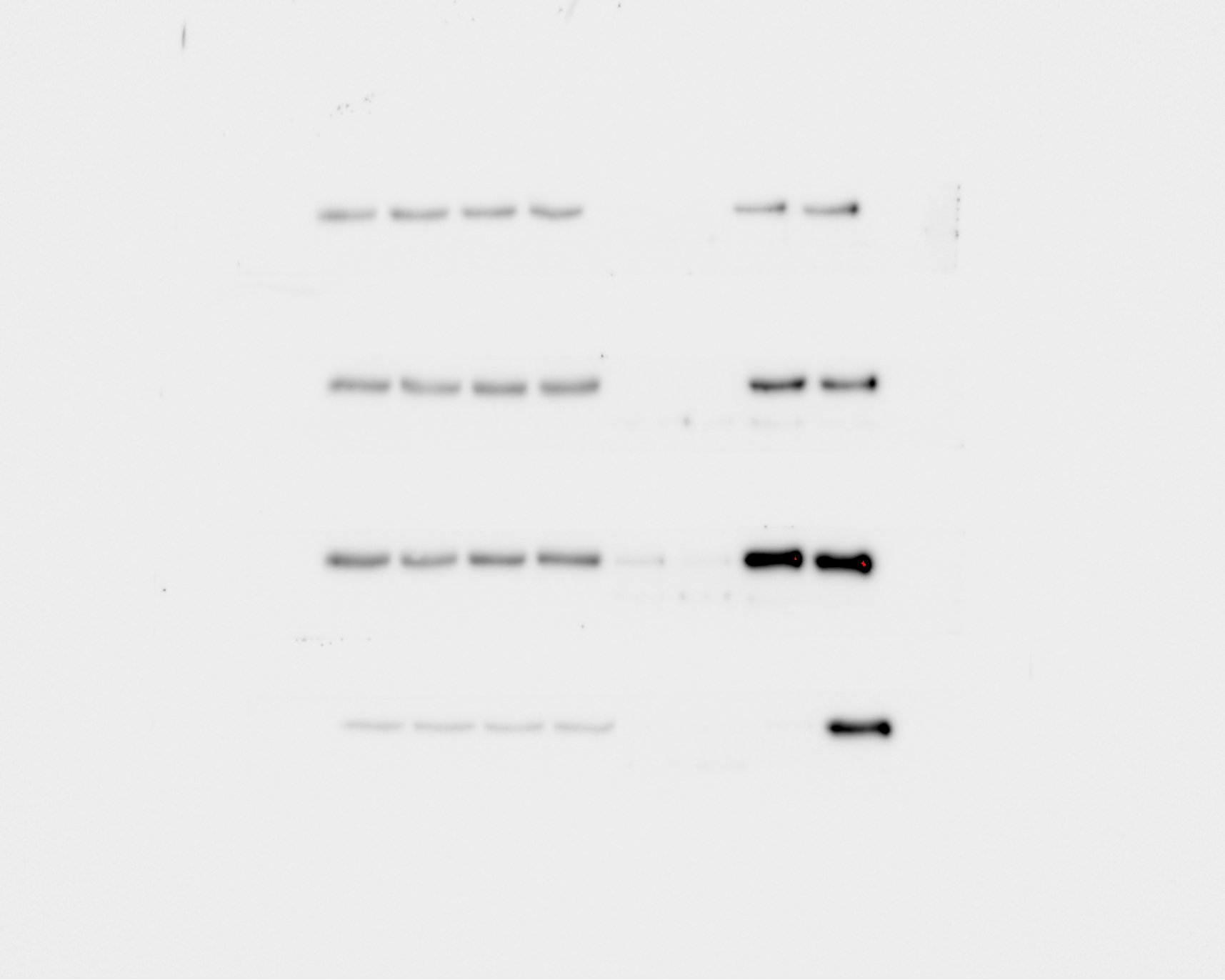

Supplement: Figure 1—figure supplement 1—source data 1. [file elife-92110-fig1-figsupp1-data1.zip › user 2021-05-21 13h04m05s(Chemiluminescence).jpg]

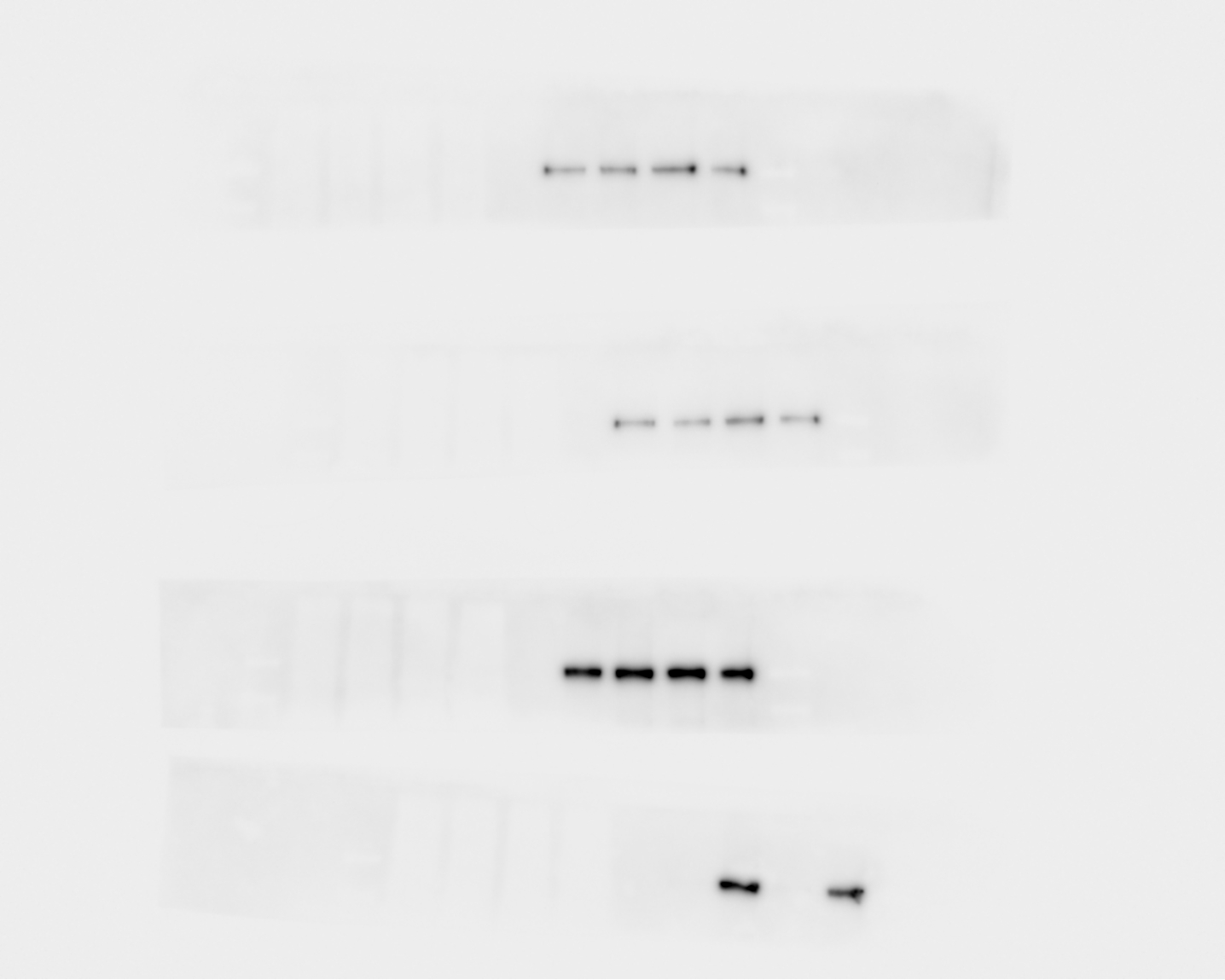

Supplement: Figure 1—figure supplement 1—source data 1. [file elife-92110-fig1-figsupp1-data1.zip › user 2021-05-26 10h01m30s(Chemiluminescence).jpg]

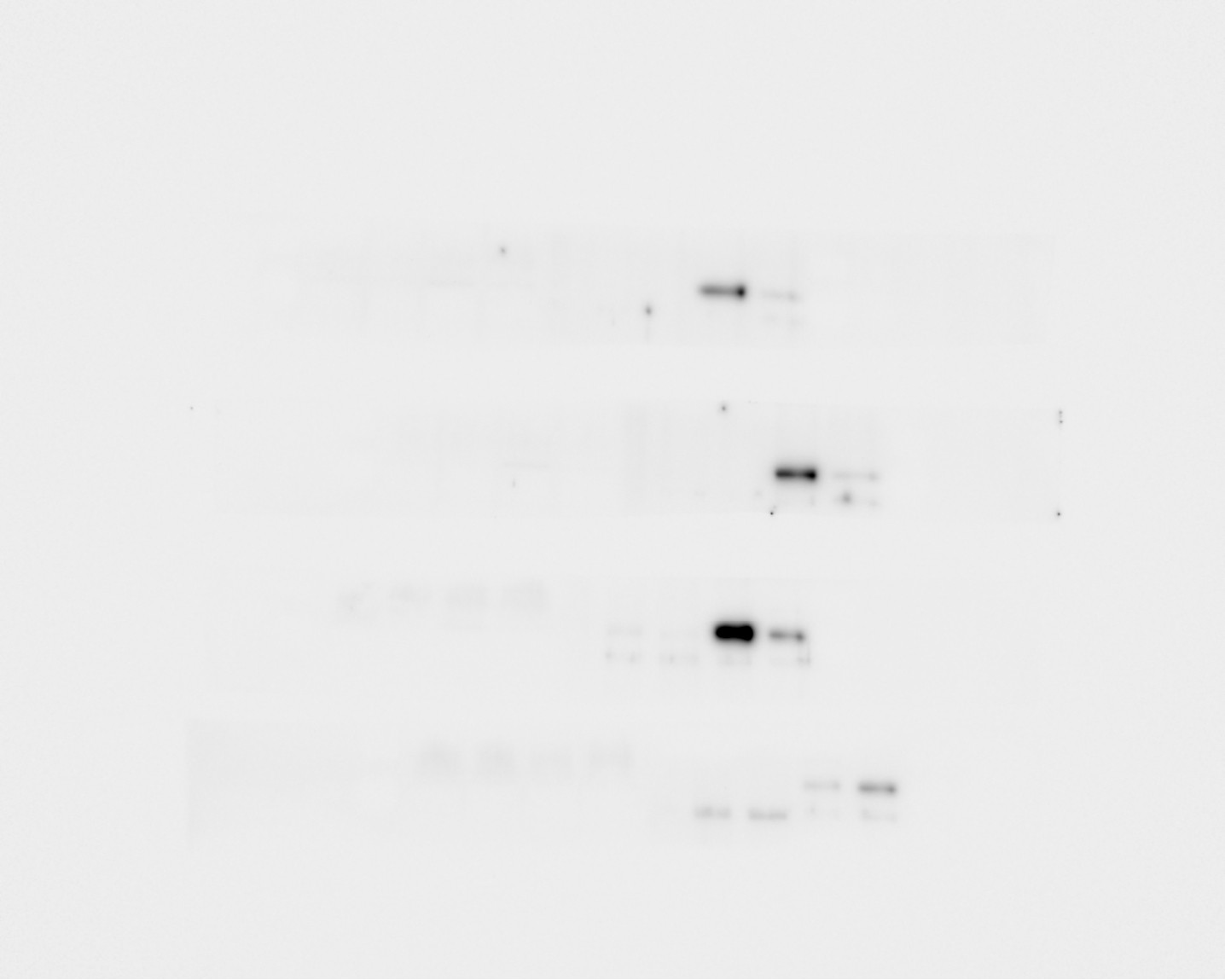

Supplement: Figure 1—figure supplement 1—source data 1. [file elife-92110-fig1-figsupp1-data1.zip › user 2021-05-26 11h35m48s(Chemiluminescence).jpg]

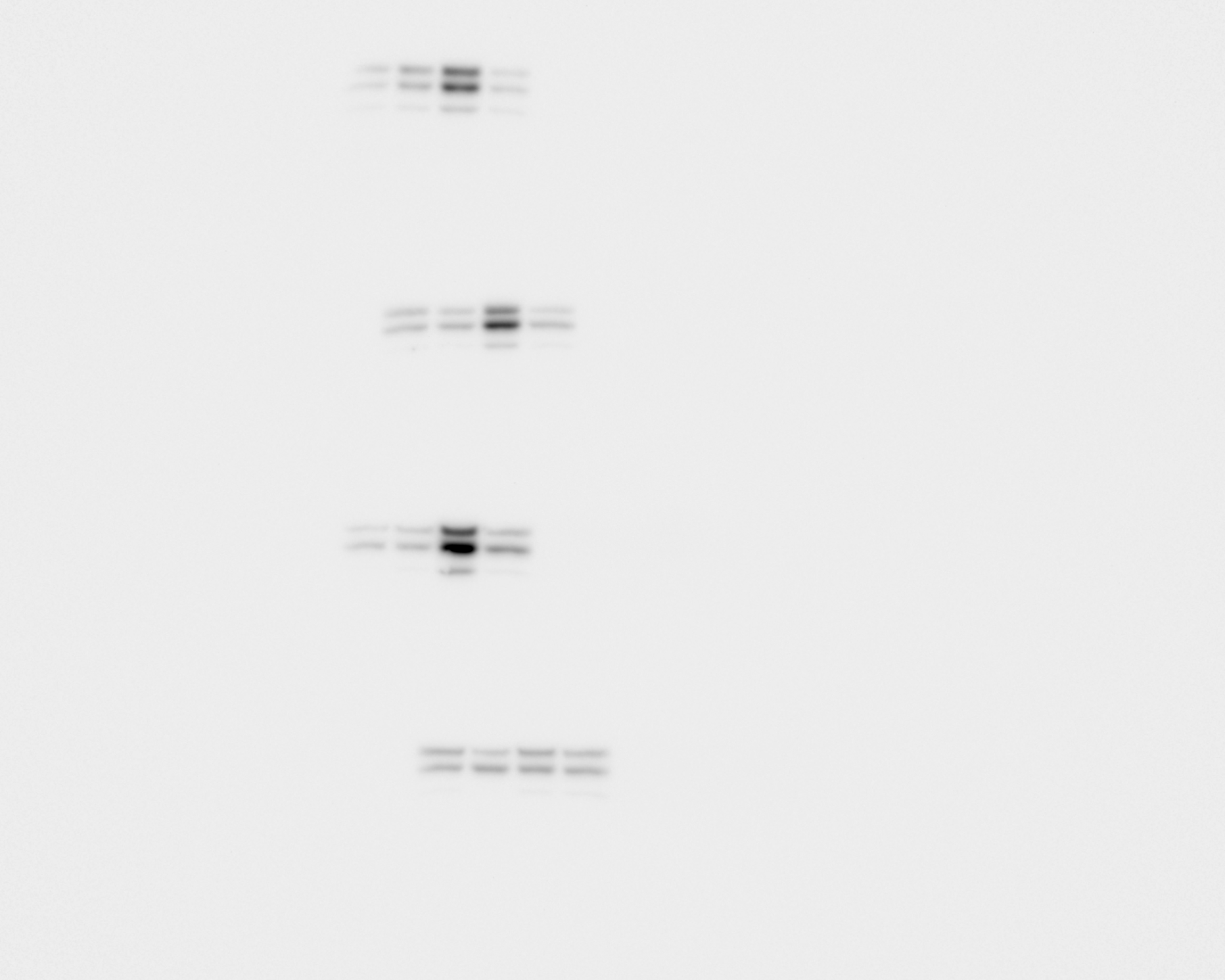

Supplement: Figure 1—figure supplement 1—source data 1. [file elife-92110-fig1-figsupp1-data1.zip › user 2021-05-26 12h22m44s(Chemiluminescence).jpg]

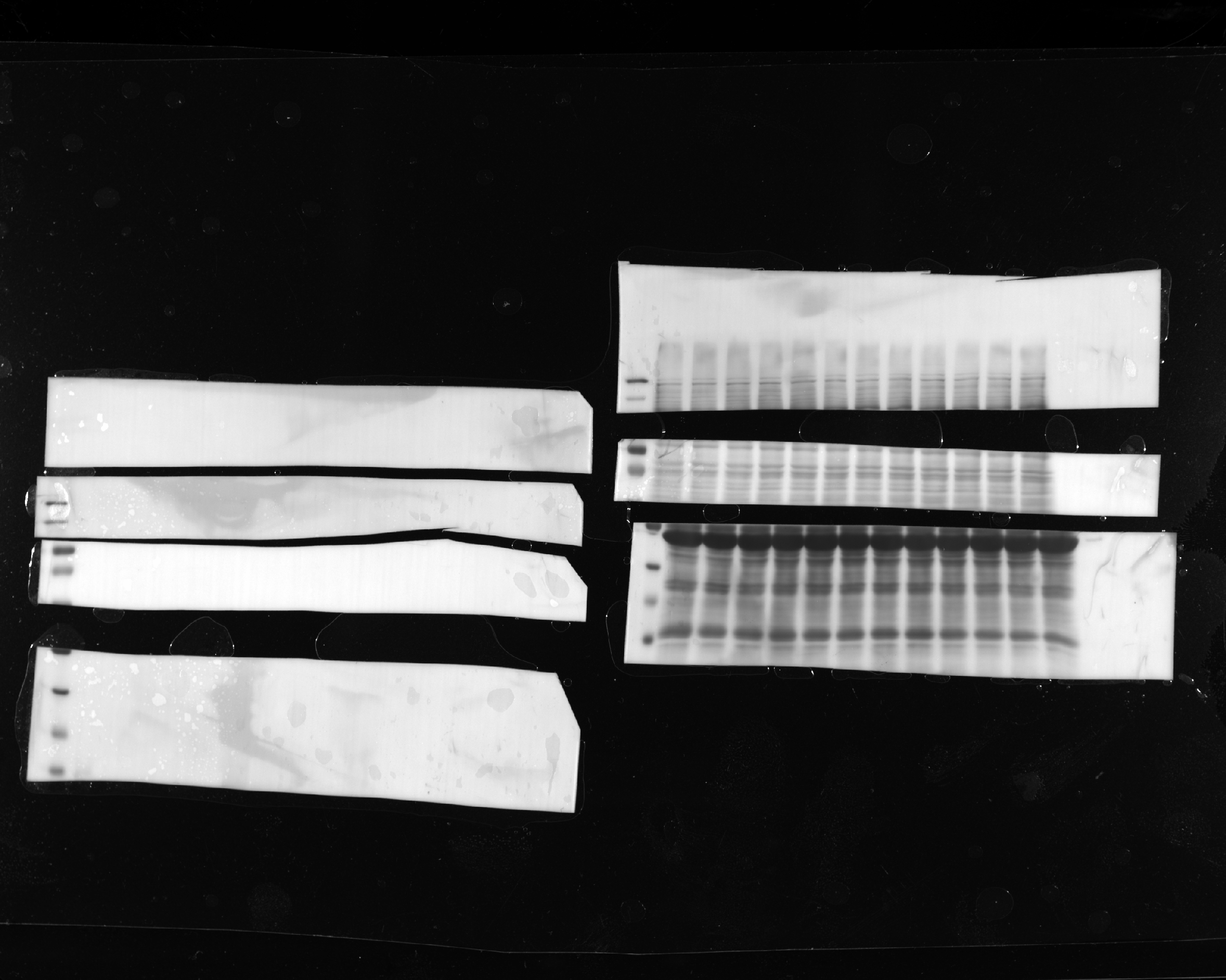

Supplement: Figure 3—source data 1. [file elife-92110-fig3-data1.zip › user 2023-06-13 15h55m44s(Colorimetric)_CBBG.jpg]

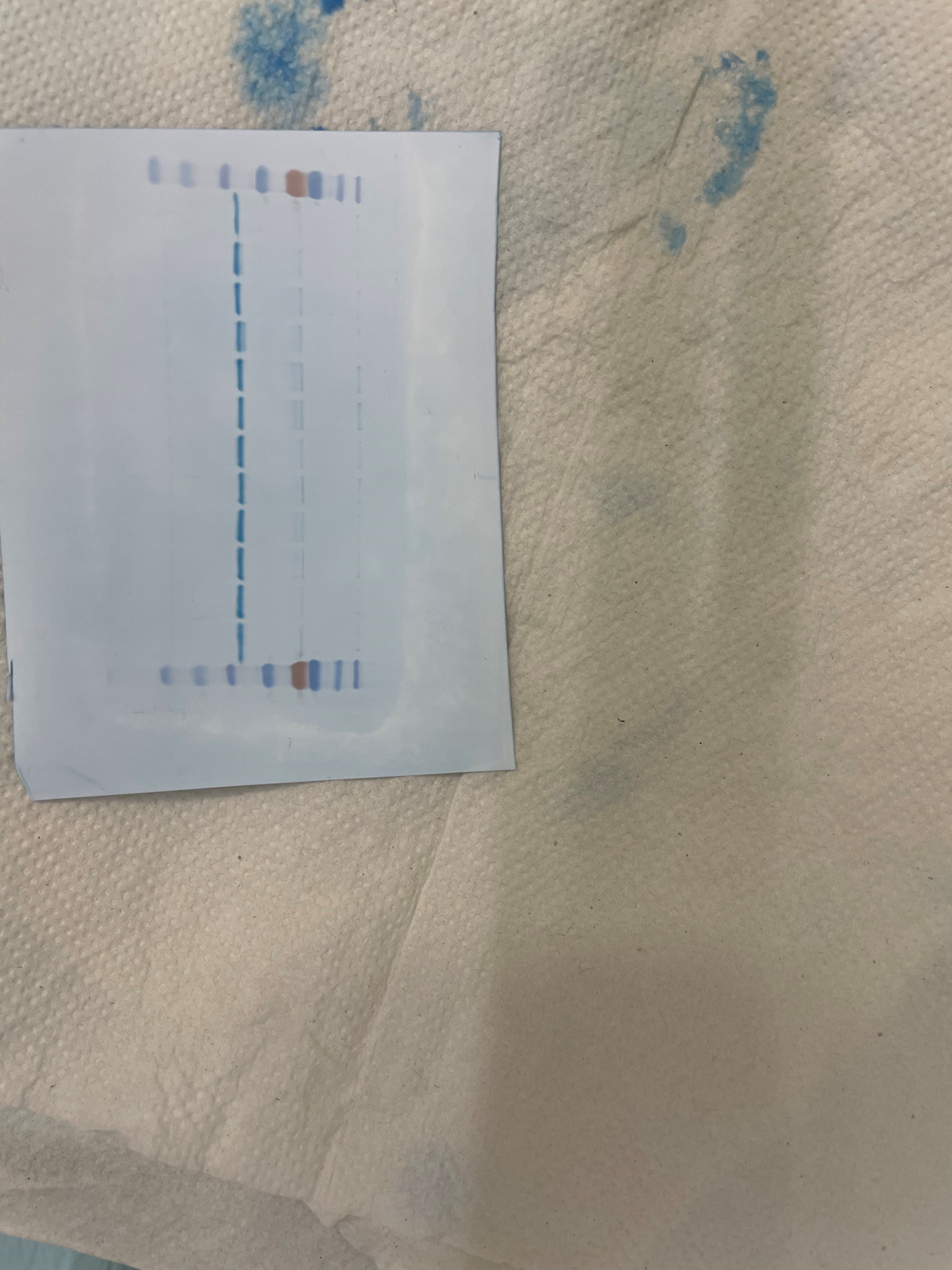

Supplement: Figure 3—source data 1. [file elife-92110-fig3-data1.zip › 2023-06-05_IPkinase_exp4_CBBG.jpg]

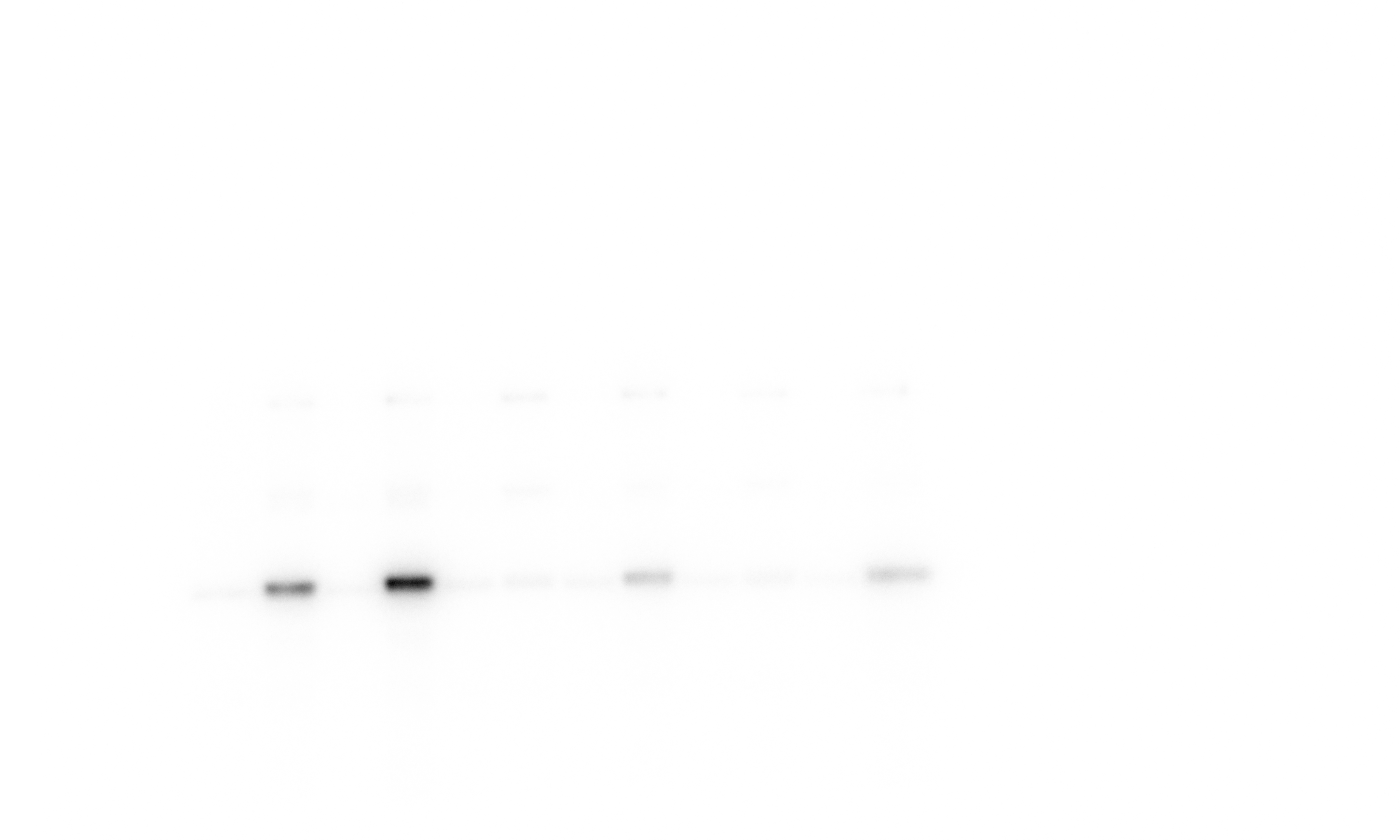

Supplement: Figure 3—source data 1. [file elife-92110-fig3-data1.zip › 20230606-123658-[Phosphor].tif]

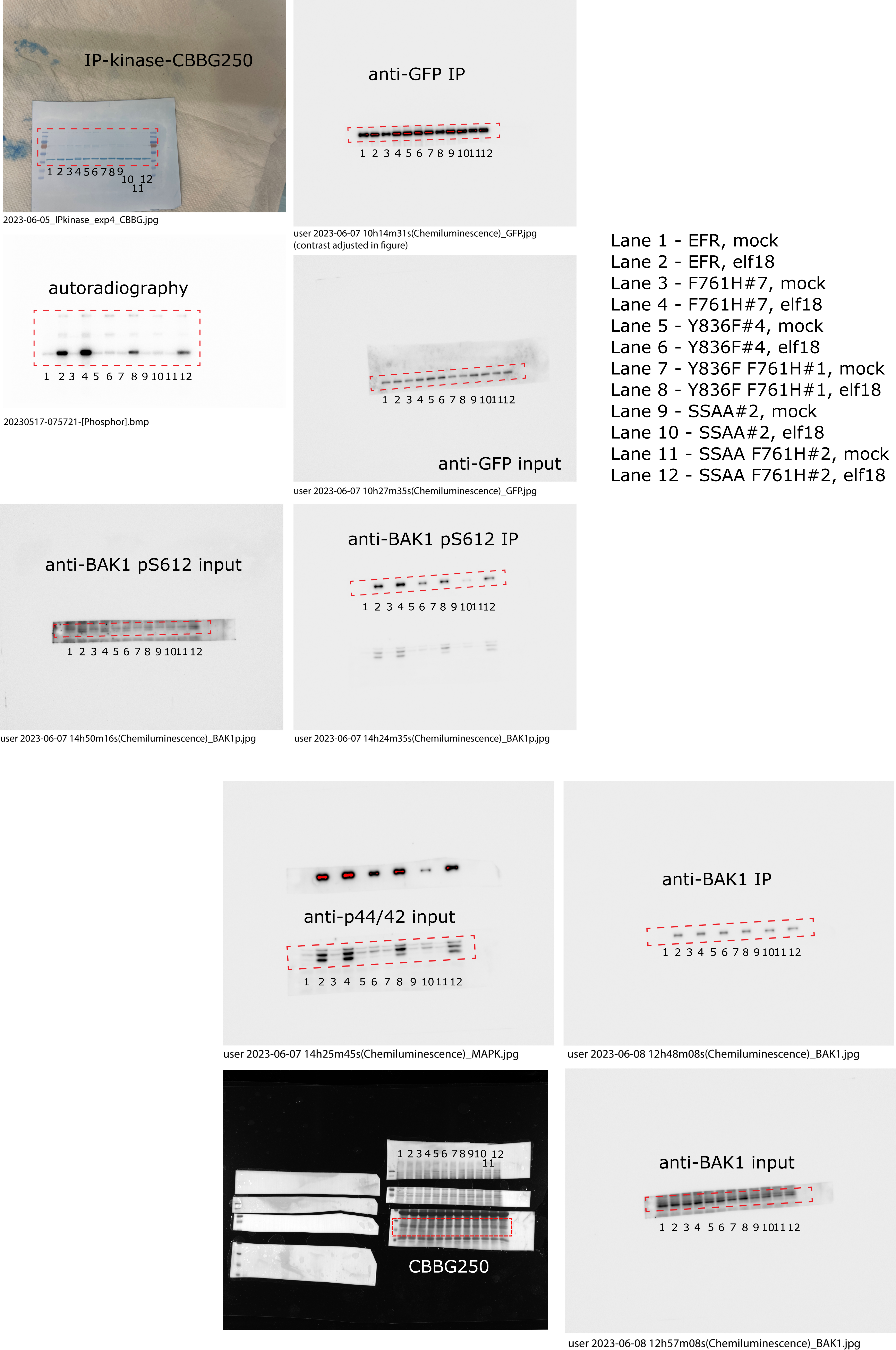

Supplement: Figure 3—source data 1. [file elife-92110-fig3-data1.zip › annotated.png]

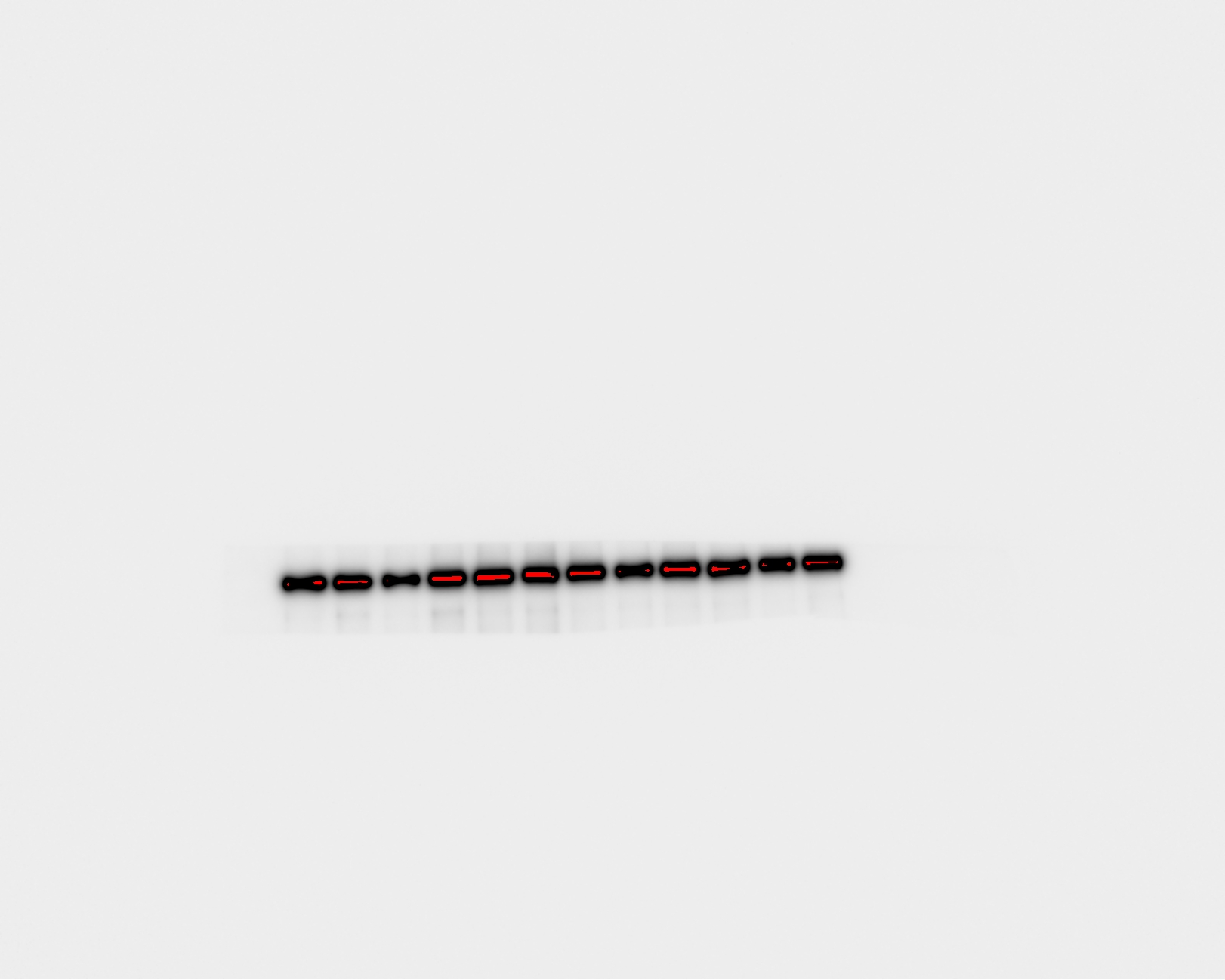

Supplement: Figure 3—source data 1. [file elife-92110-fig3-data1.zip › user 2023-06-07 10h14m31s(Chemiluminescence)_GFP.jpg]

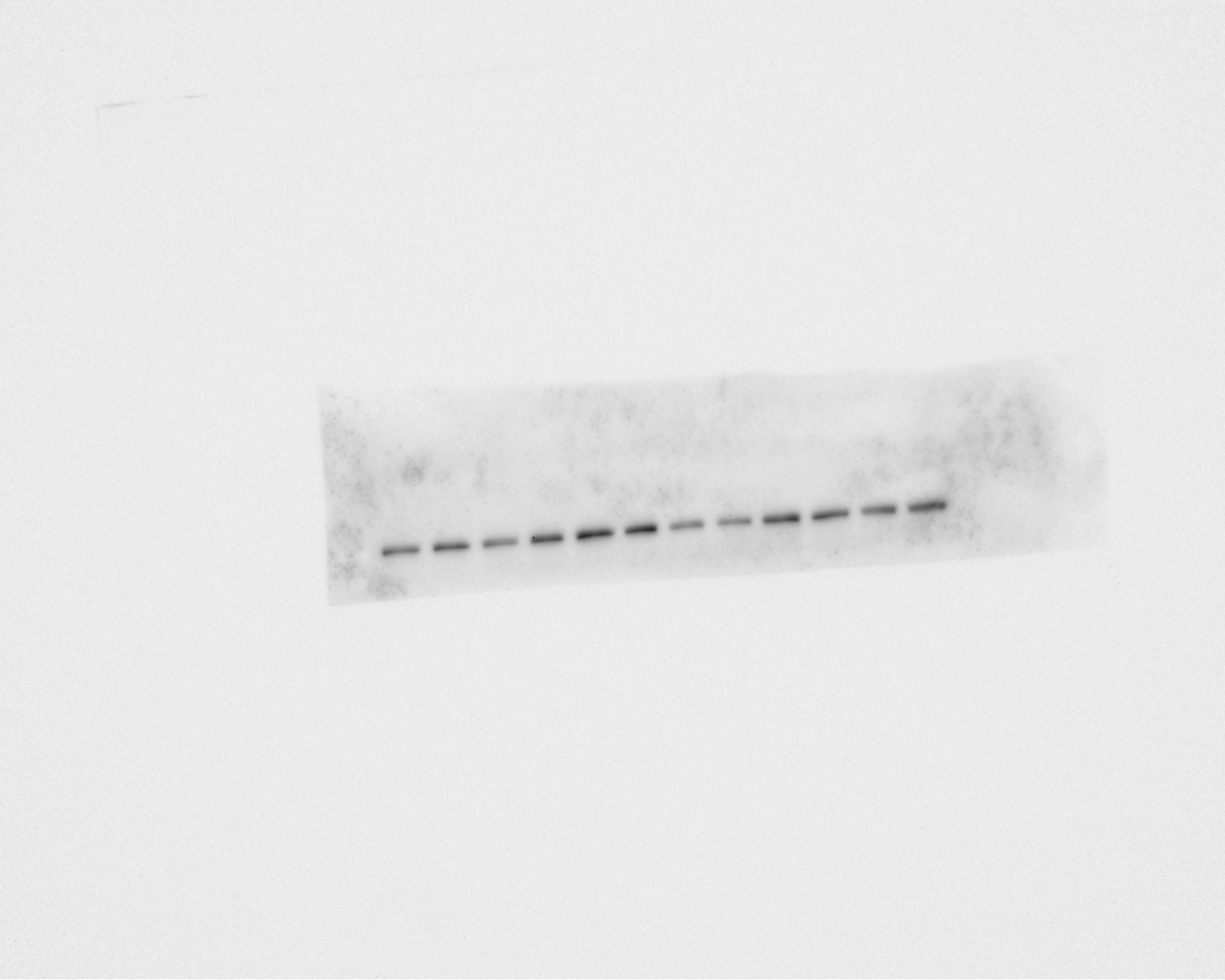

Supplement: Figure 3—source data 1. [file elife-92110-fig3-data1.zip › user 2023-06-07 10h27m35s(Chemiluminescence)_GFP.jpg]

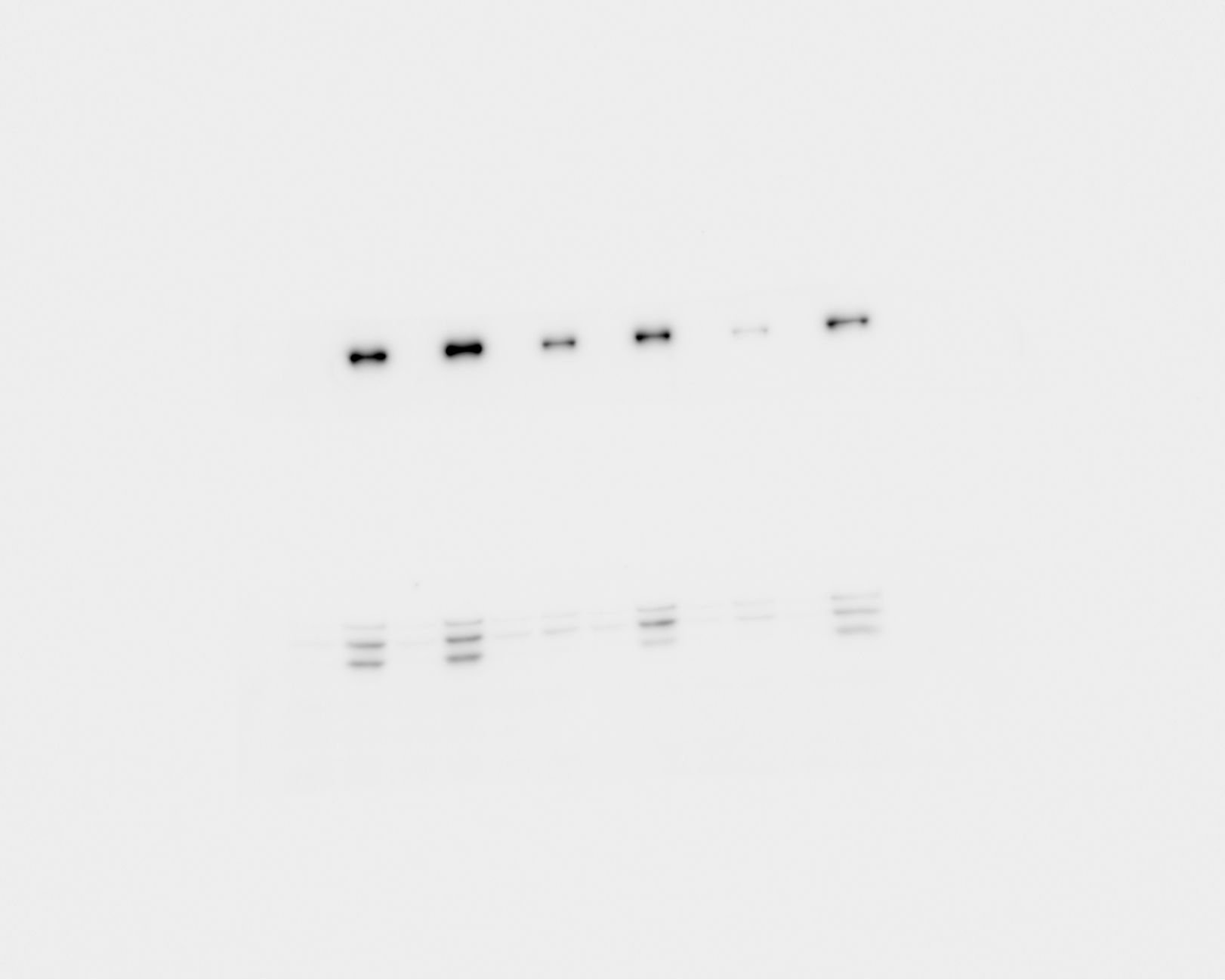

Supplement: Figure 3—source data 1. [file elife-92110-fig3-data1.zip › user 2023-06-07 14h24m35s(Chemiluminescence)_BAK1p.jpg]

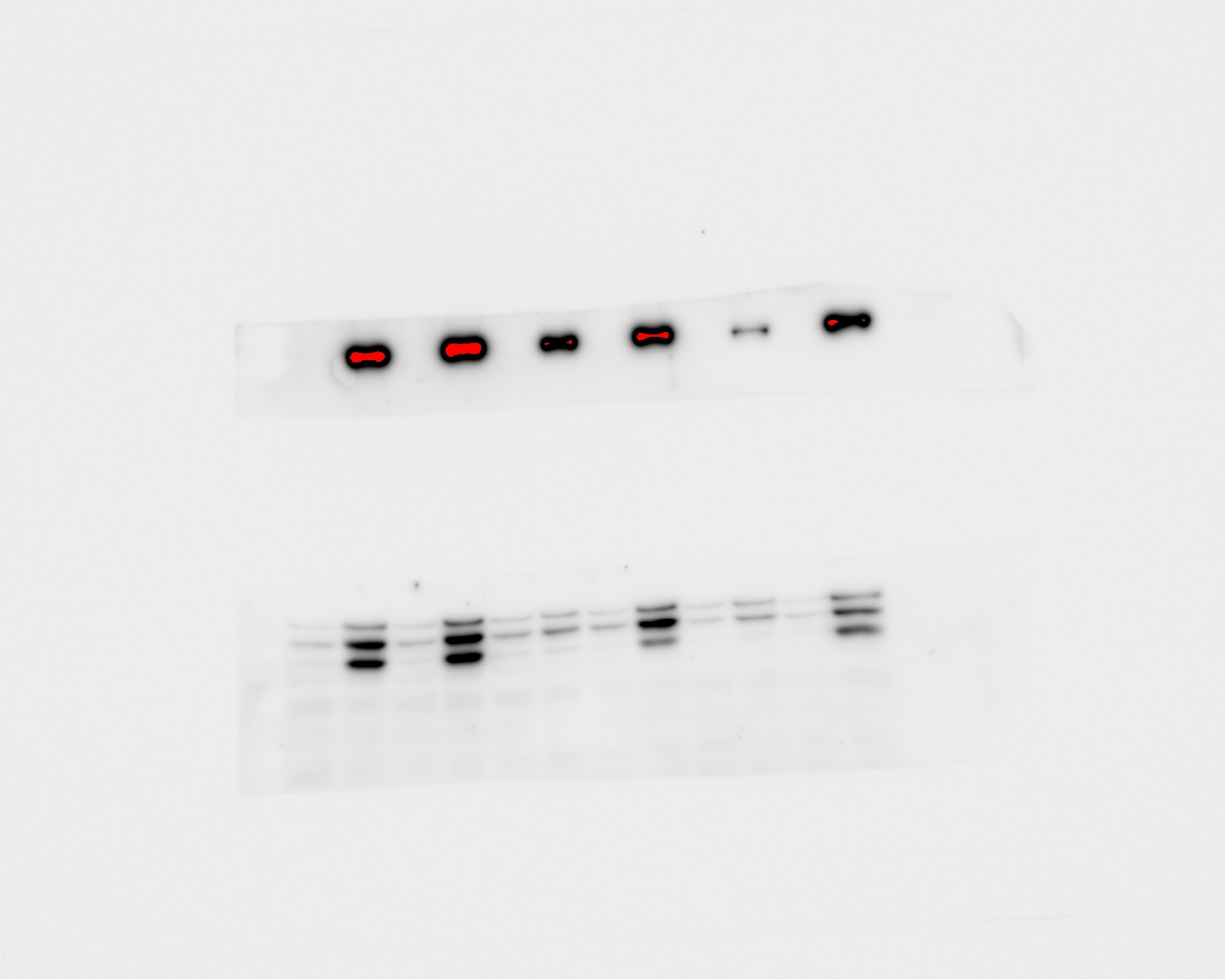

Supplement: Figure 3—source data 1. [file elife-92110-fig3-data1.zip › user 2023-06-07 14h25m45s(Chemiluminescence)_MAPK.jpg]

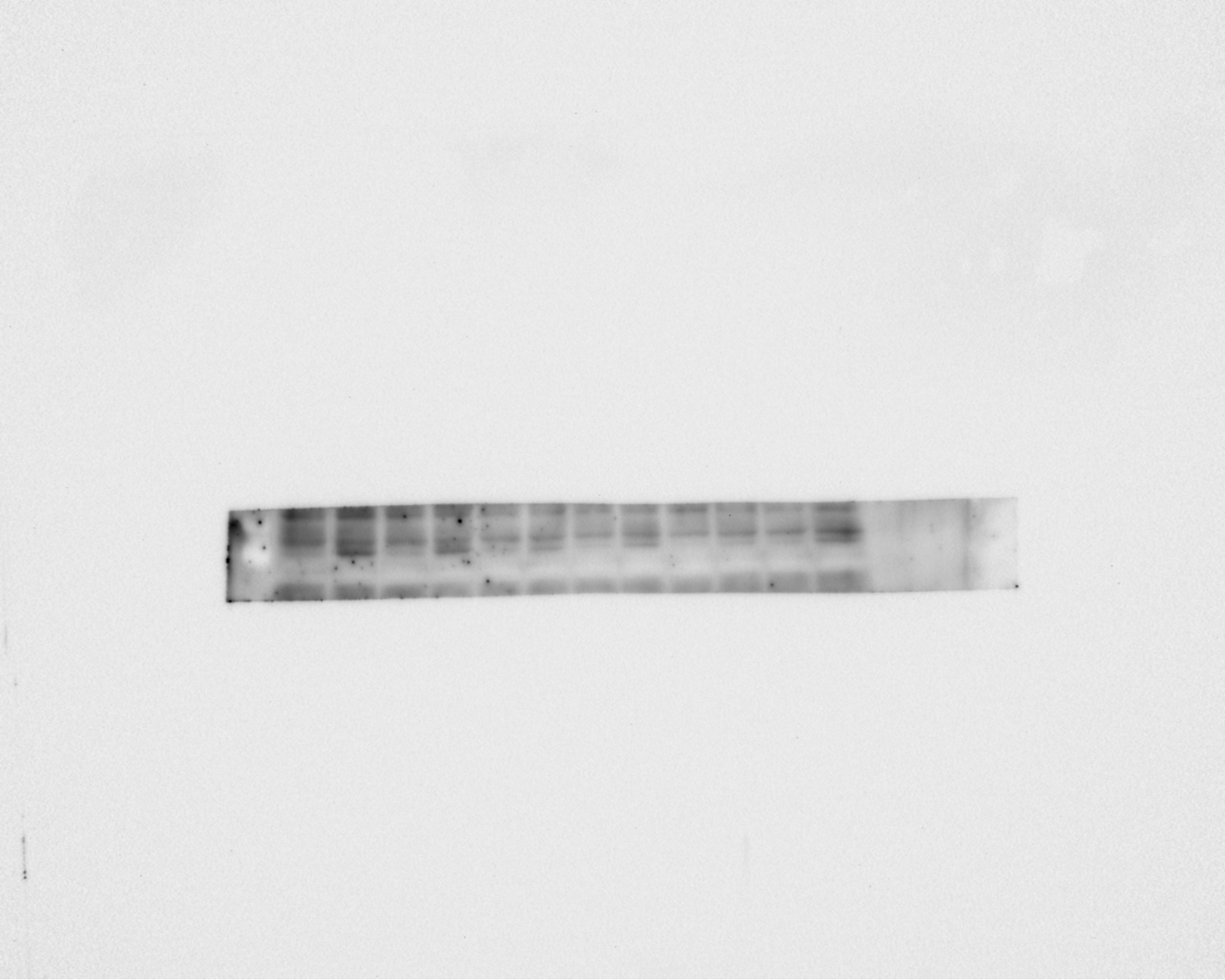

Supplement: Figure 3—source data 1. [file elife-92110-fig3-data1.zip › user 2023-06-07 14h50m16s(Chemiluminescence)_BAK1p.jpg]

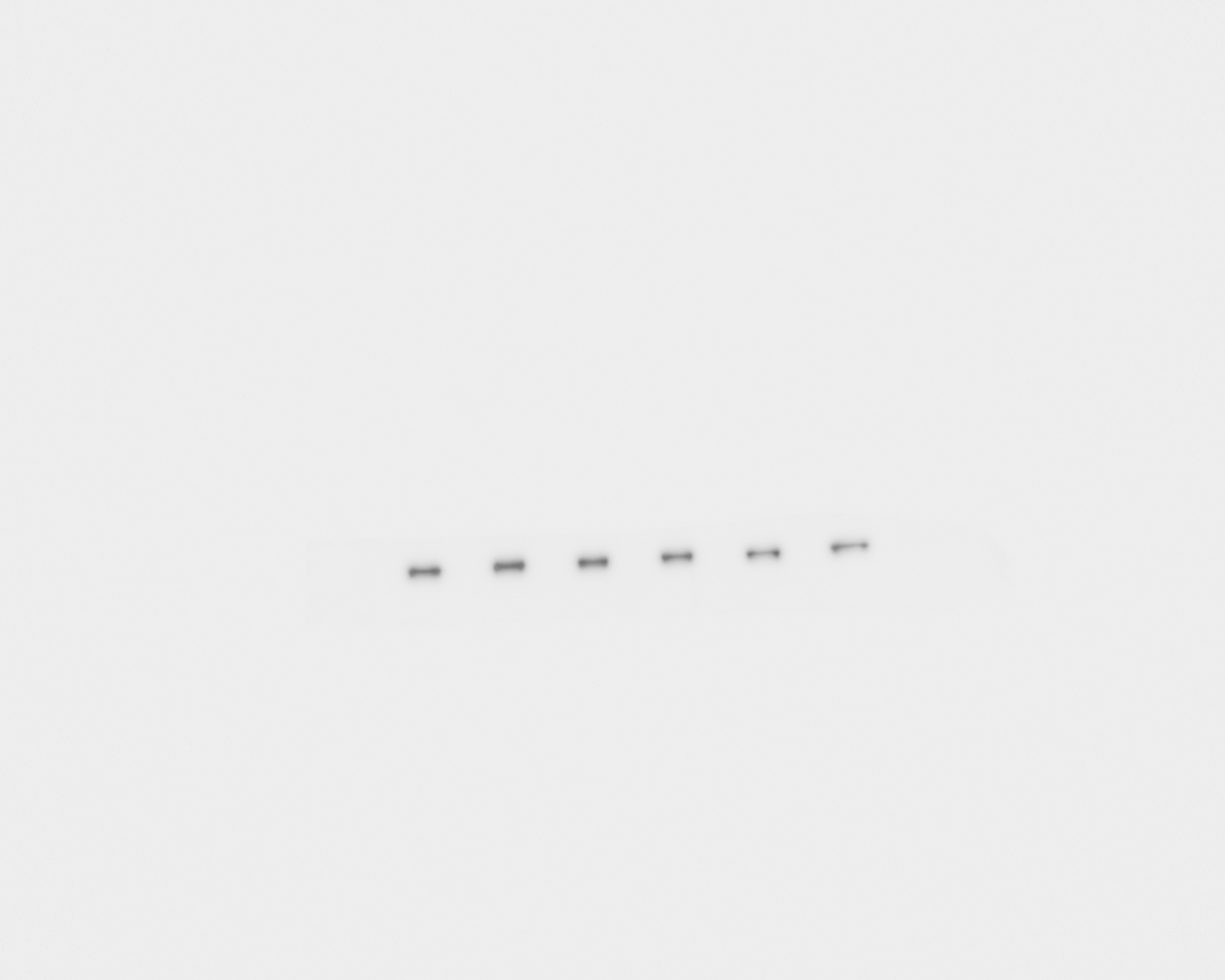

Supplement: Figure 3—source data 1. [file elife-92110-fig3-data1.zip › user 2023-06-08 12h48m08s(Chemiluminescence)_BAK1.jpg]

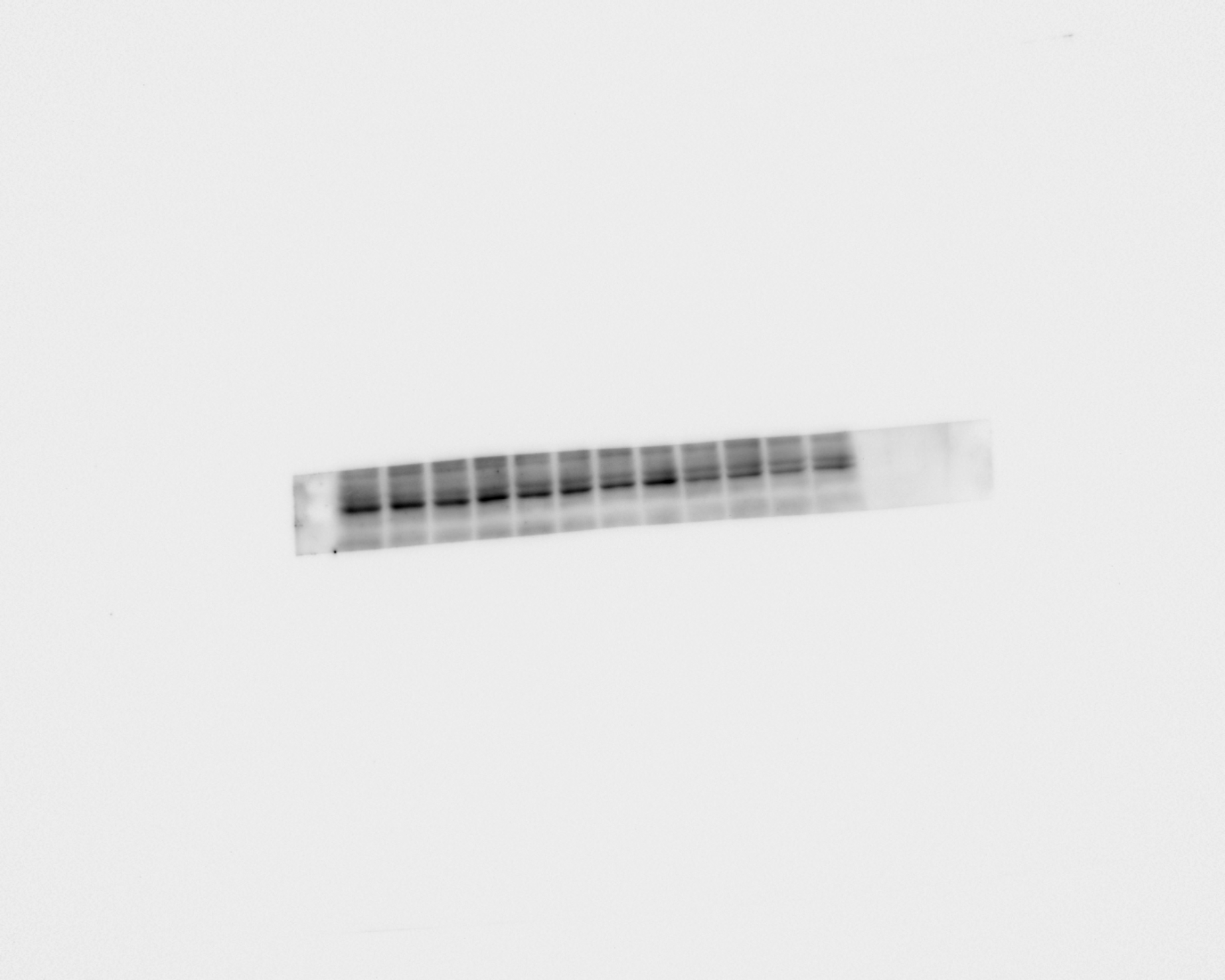

Supplement: Figure 3—source data 1. [file elife-92110-fig3-data1.zip › user 2023-06-08 12h57m08s(Chemiluminescence)_BAK1.jpg]

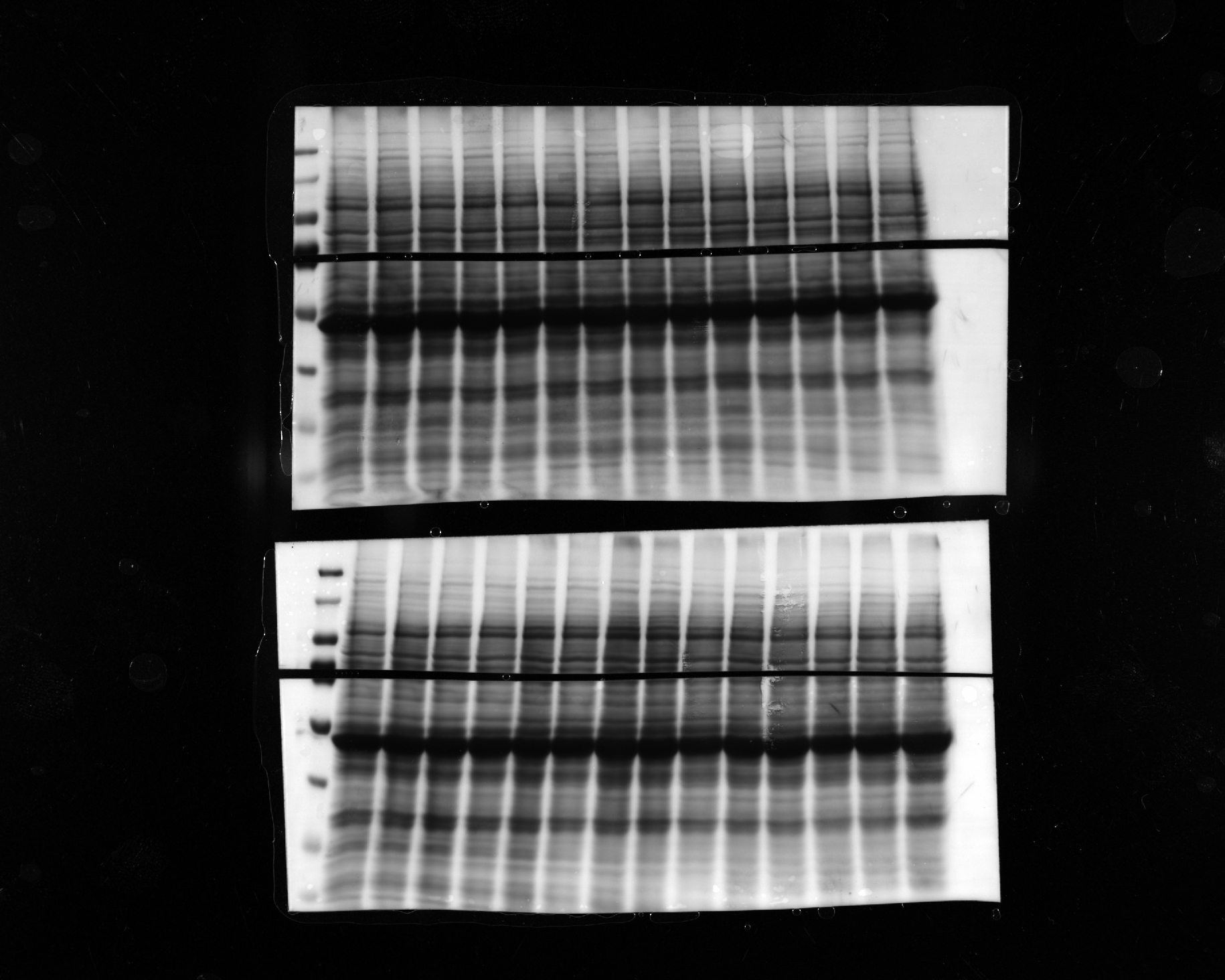

Supplement: Figure 3—figure supplement 1—source data 1. [file elife-92110-fig3-figsupp1-data1.zip › user 2023-02-08 16h04m55s(Colorimetric)_CBBG.jpg]

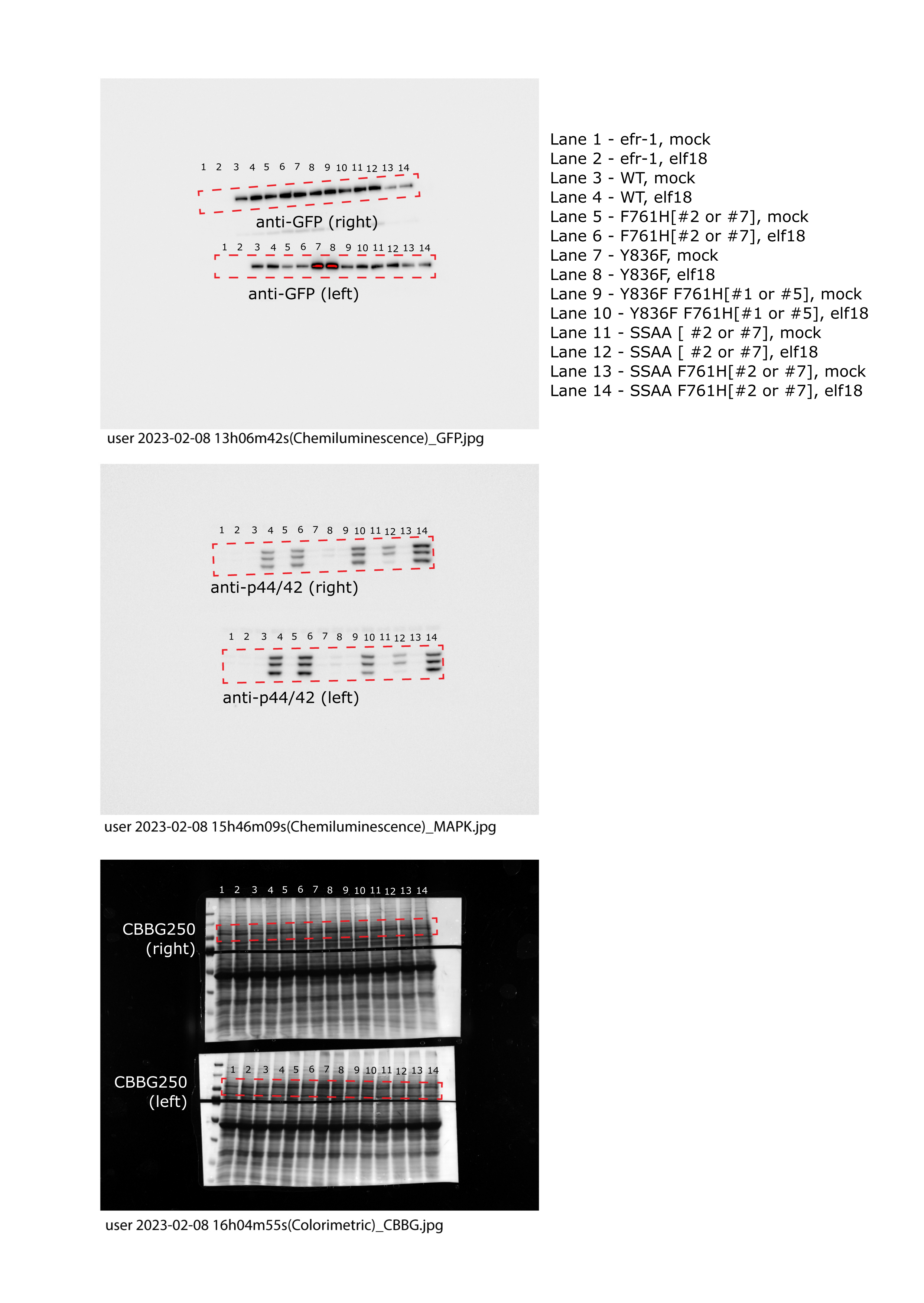

Supplement: Figure 3—figure supplement 1—source data 1. [file elife-92110-fig3-figsupp1-data1.zip › annotated.png]

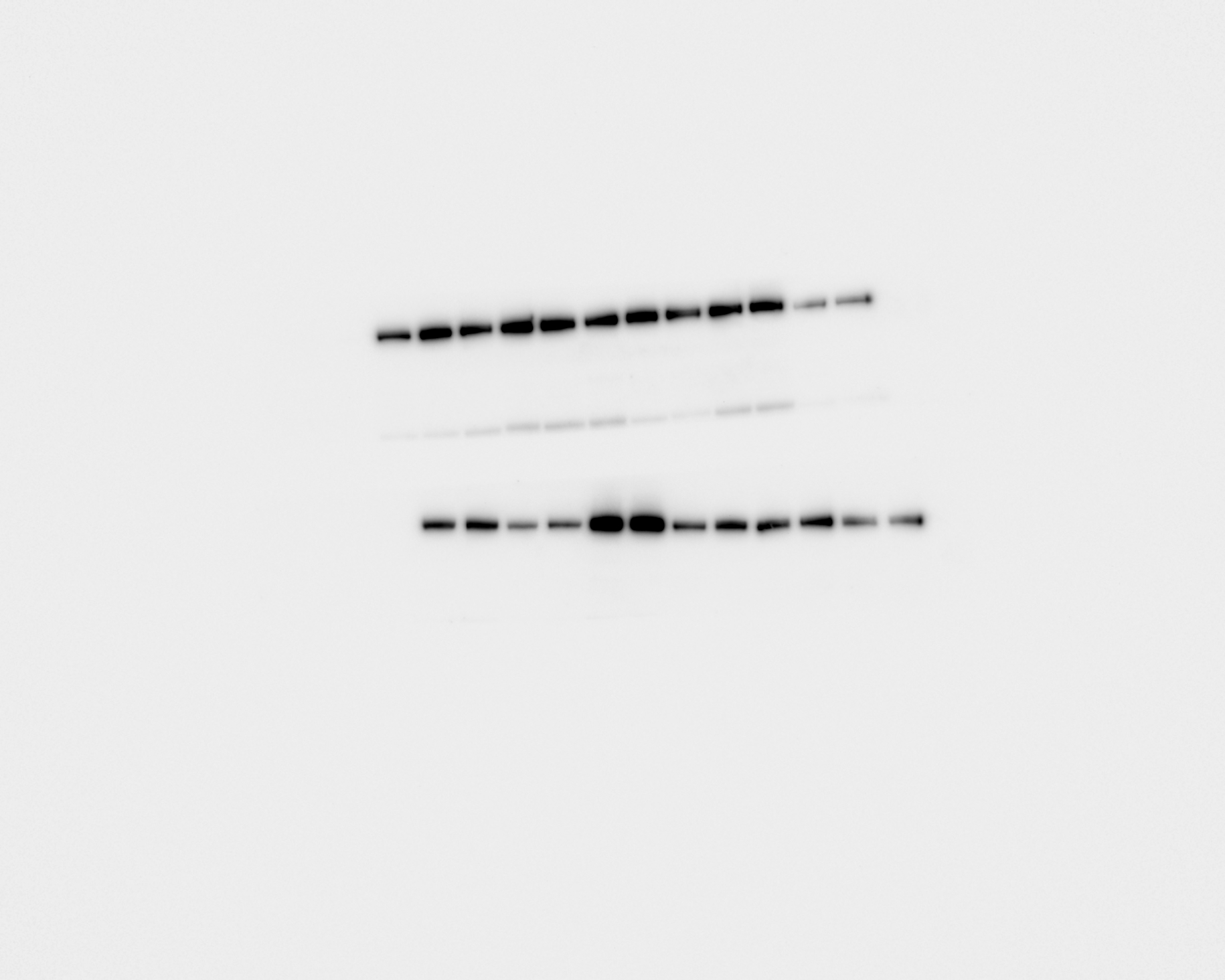

Supplement: Figure 3—figure supplement 1—source data 1. [file elife-92110-fig3-figsupp1-data1.zip › user 2023-02-08 13h06m42s(Chemiluminescence)_GFP2.jpg]

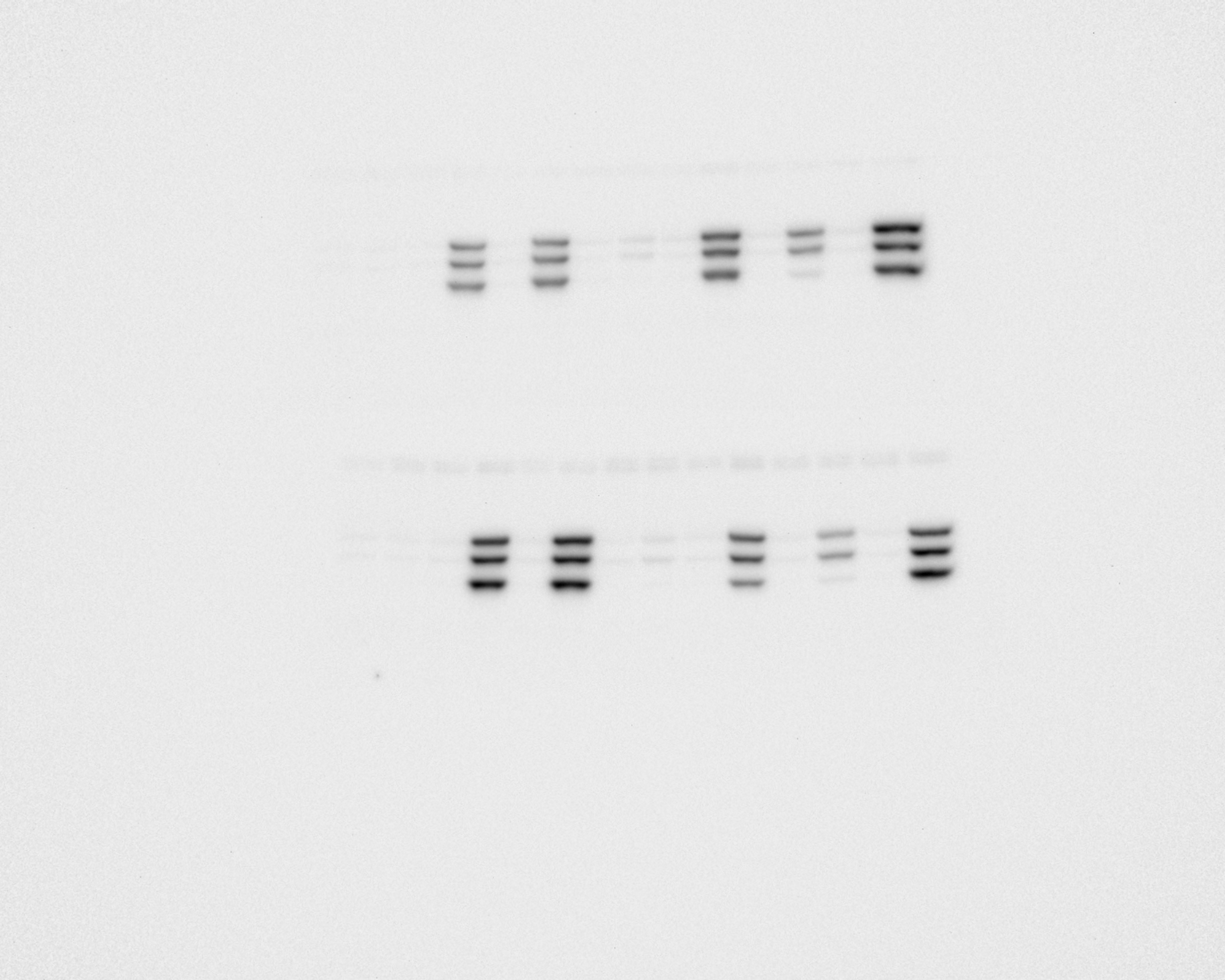

Supplement: Figure 3—figure supplement 1—source data 1. [file elife-92110-fig3-figsupp1-data1.zip › user 2023-02-08 15h46m09s(Chemiluminescence)_MAPK.jpg]

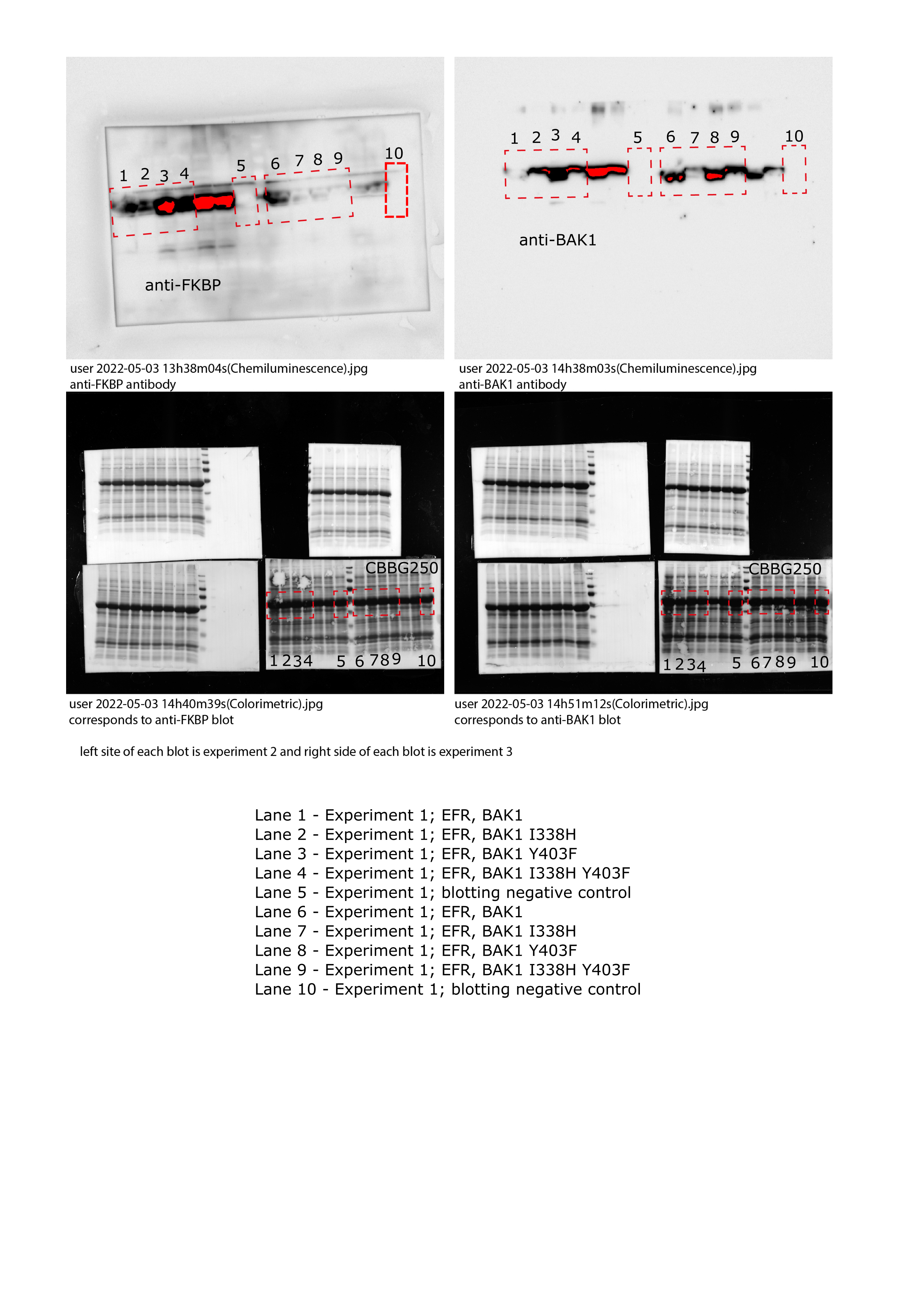

Supplement: Figure 4—figure supplement 1—source data 1. [file elife-92110-fig4-figsupp1-data1.zip › annotated.png]

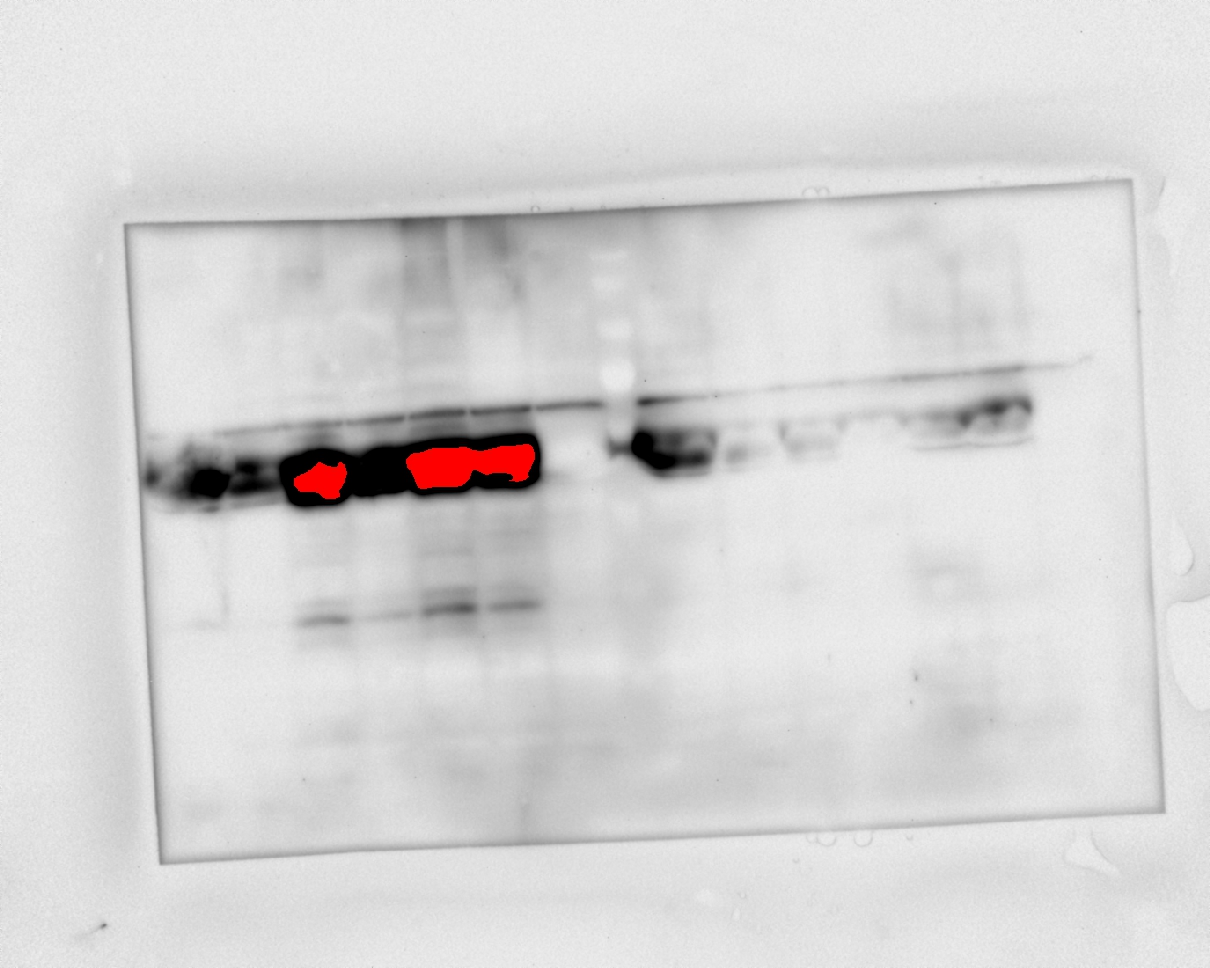

Supplement: Figure 4—figure supplement 1—source data 1. [file elife-92110-fig4-figsupp1-data1.zip › user 2022-05-03 13h38m04s(Chemiluminescence).jpg]

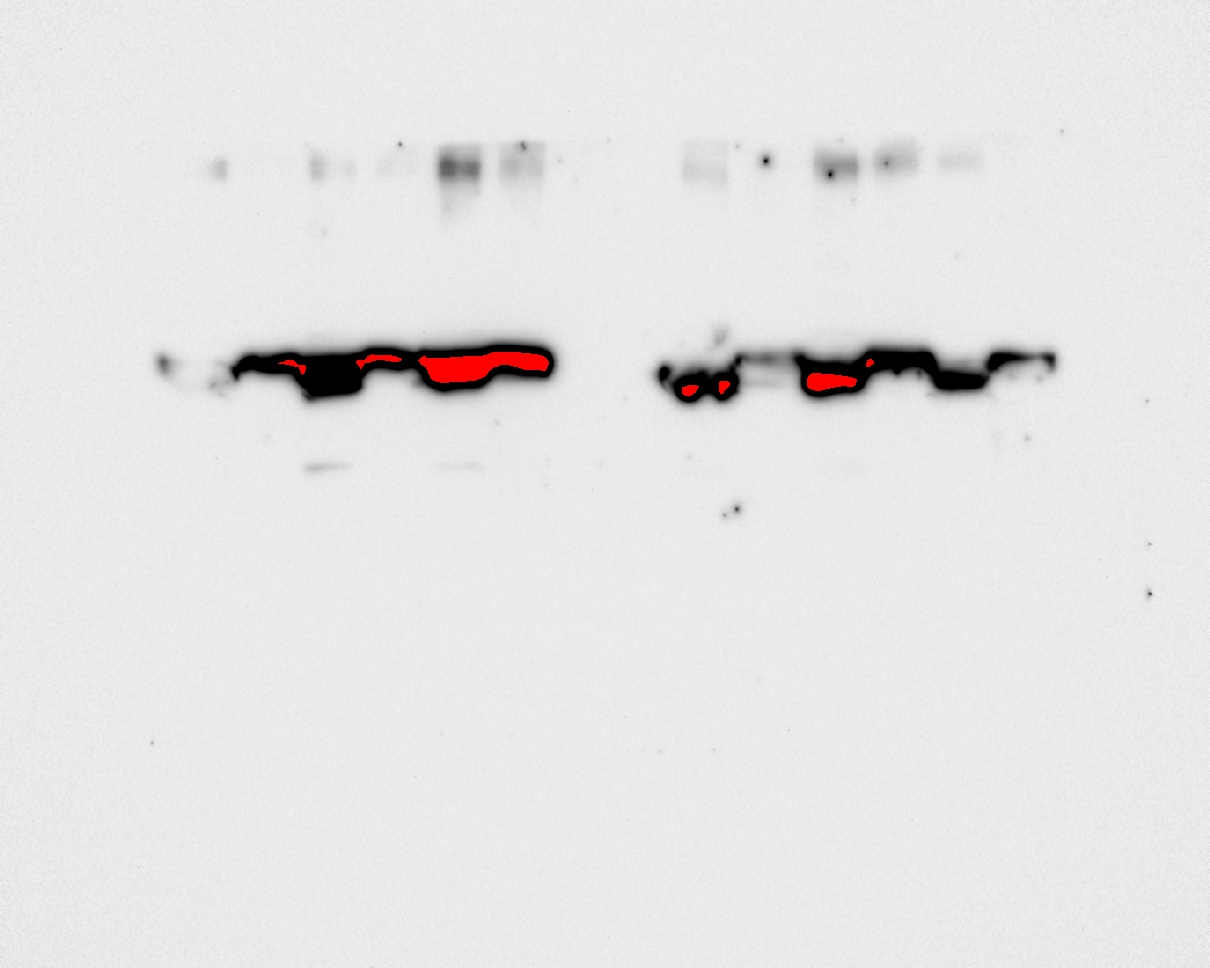

Supplement: Figure 4—figure supplement 1—source data 1. [file elife-92110-fig4-figsupp1-data1.zip › user 2022-05-03 14h38m03s(Chemiluminescence).jpg]

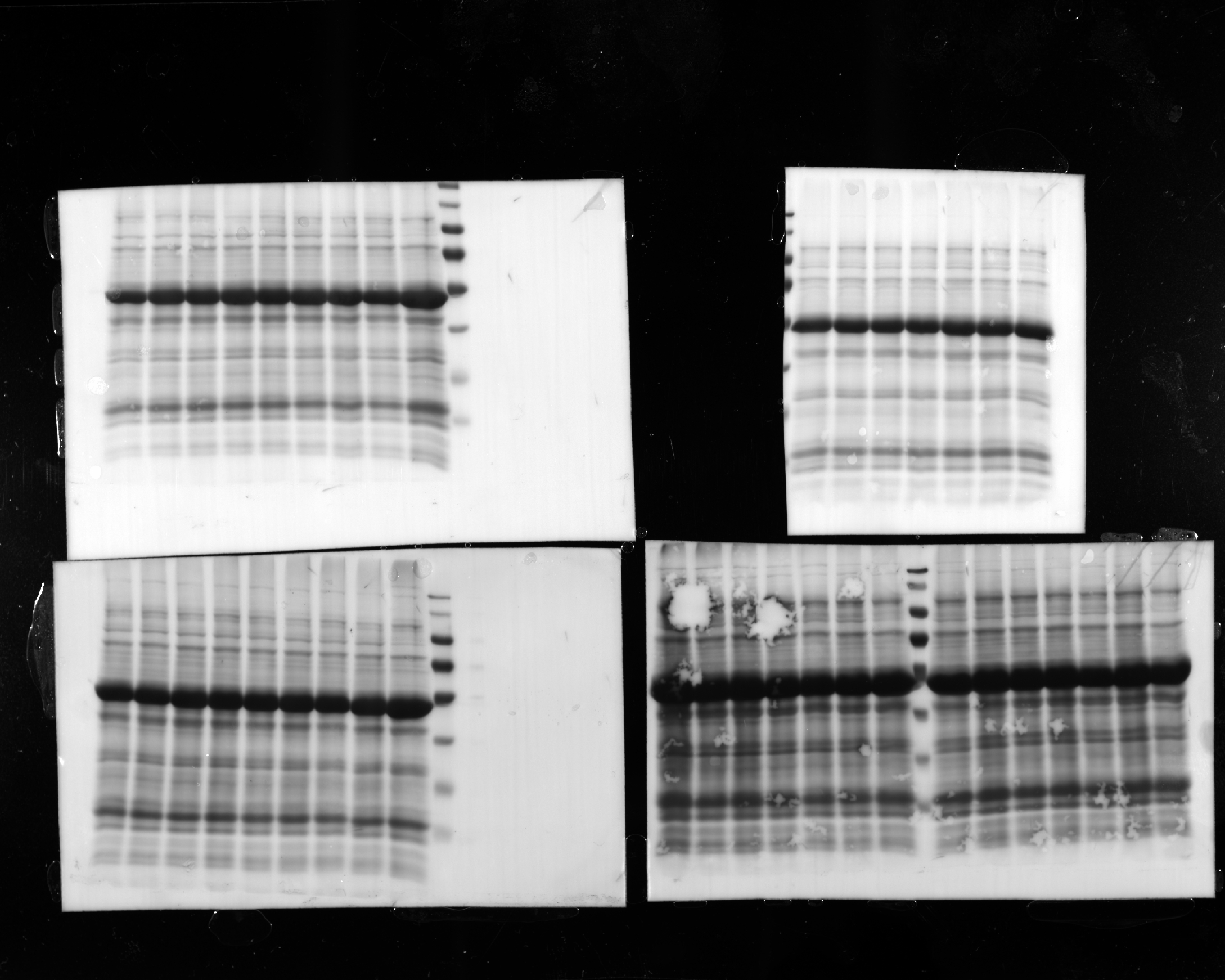

Supplement: Figure 4—figure supplement 1—source data 1. [file elife-92110-fig4-figsupp1-data1.zip › user 2022-05-03 14h40m39s(Colorimetric).jpg]

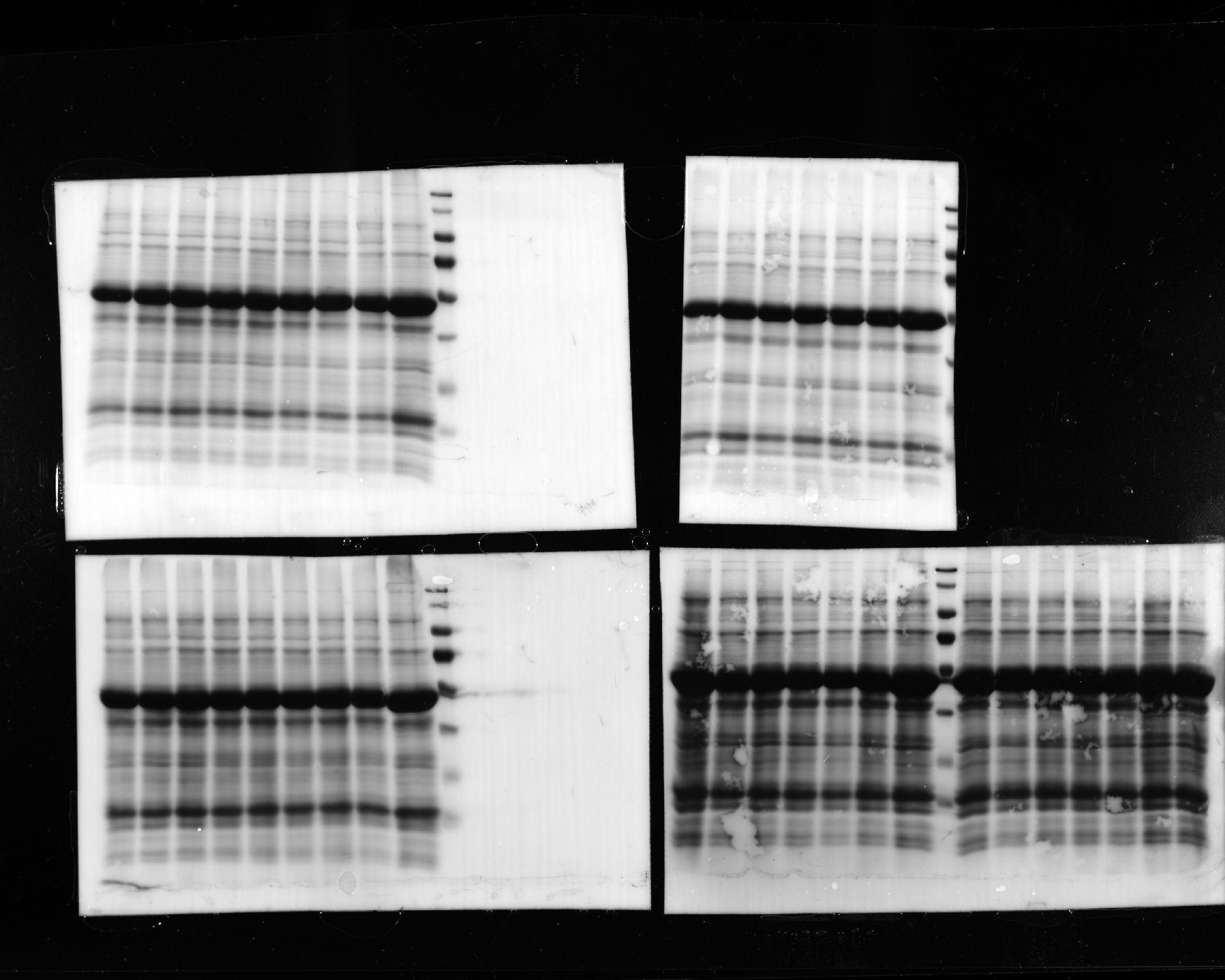

Supplement: Figure 4—figure supplement 1—source data 1. [file elife-92110-fig4-figsupp1-data1.zip › user 2022-05-03 14h51m12s(Colorimetric).jpg]

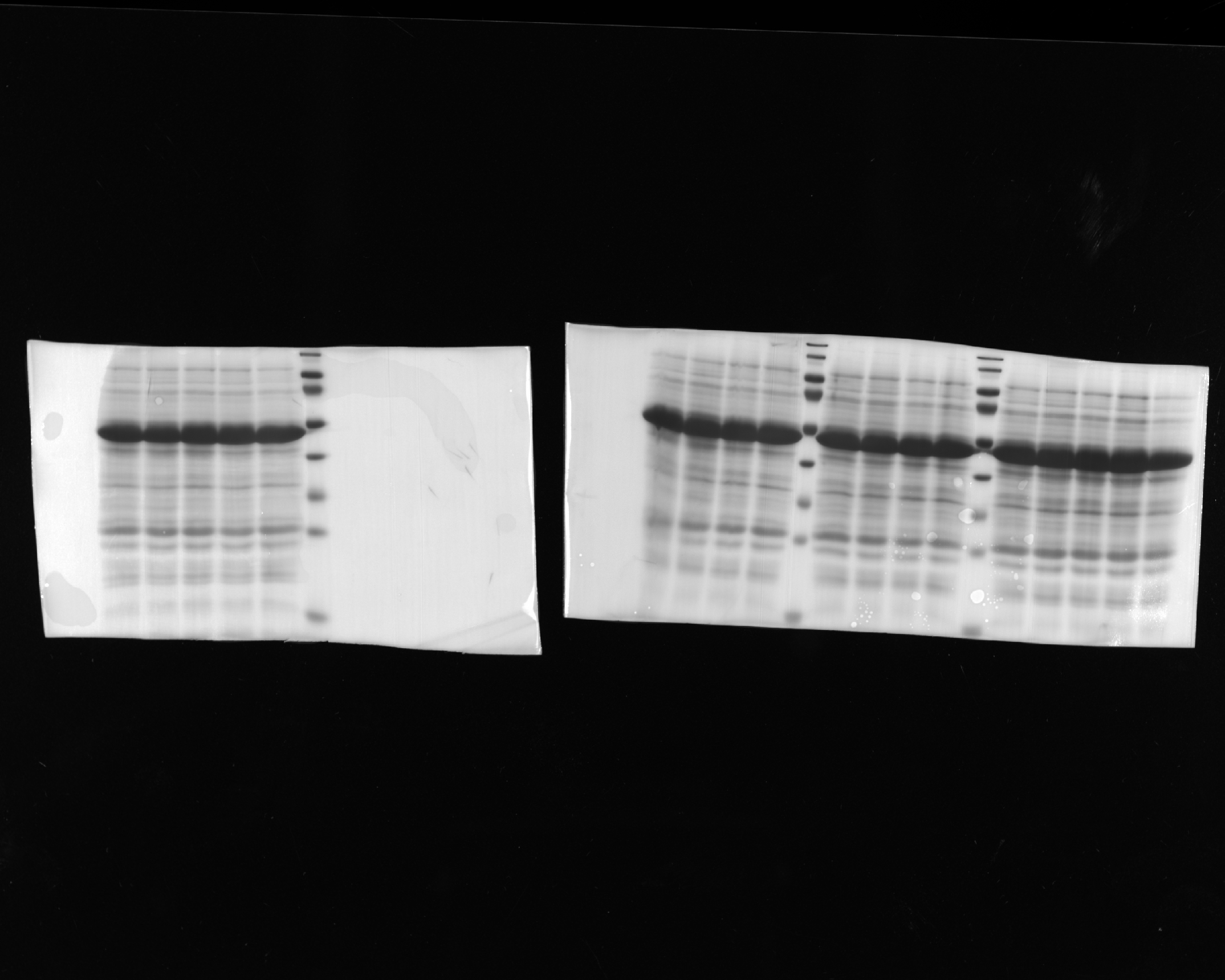

Supplement: Figure 4—figure supplement 2—source data 1. [file elife-92110-fig4-figsupp2-data1.zip › user 2023-07-19 19h30m52s(Colorimetric).jpg]

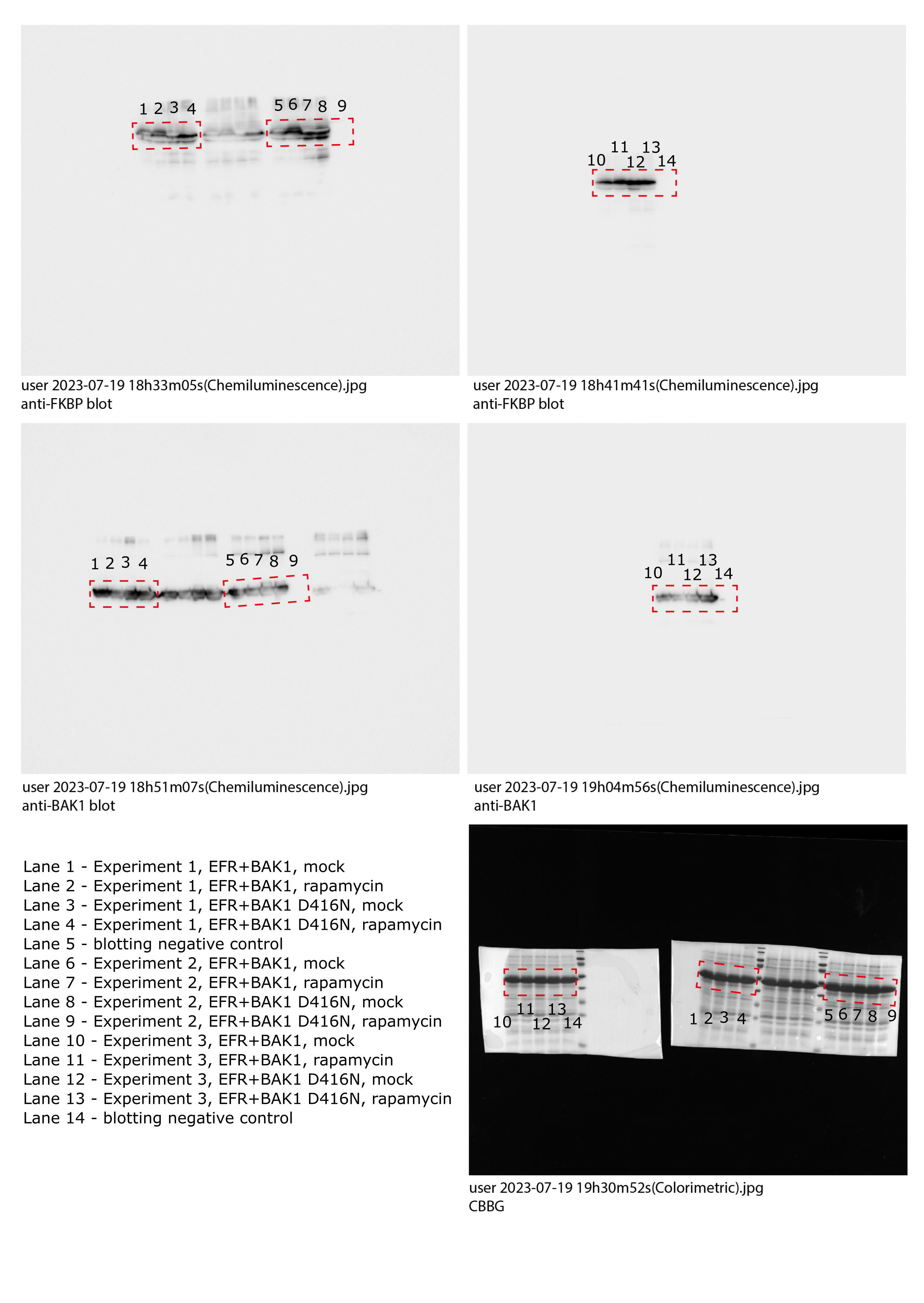

Supplement: Figure 4—figure supplement 2—source data 1. [file elife-92110-fig4-figsupp2-data1.zip › annotated.png]

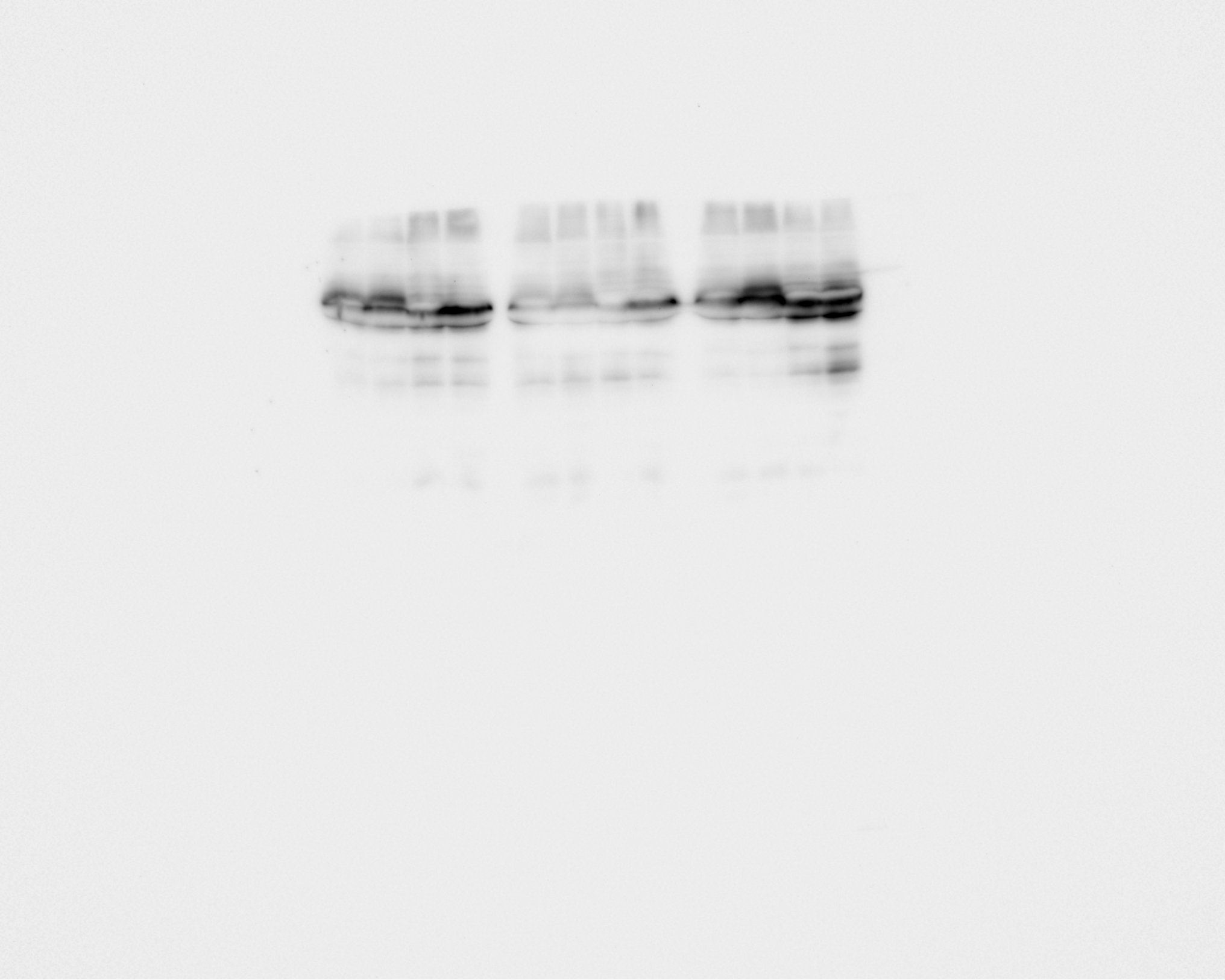

Supplement: Figure 4—figure supplement 2—source data 1. [file elife-92110-fig4-figsupp2-data1.zip › user 2023-07-19 18h33m05s(Chemiluminescence).jpg]

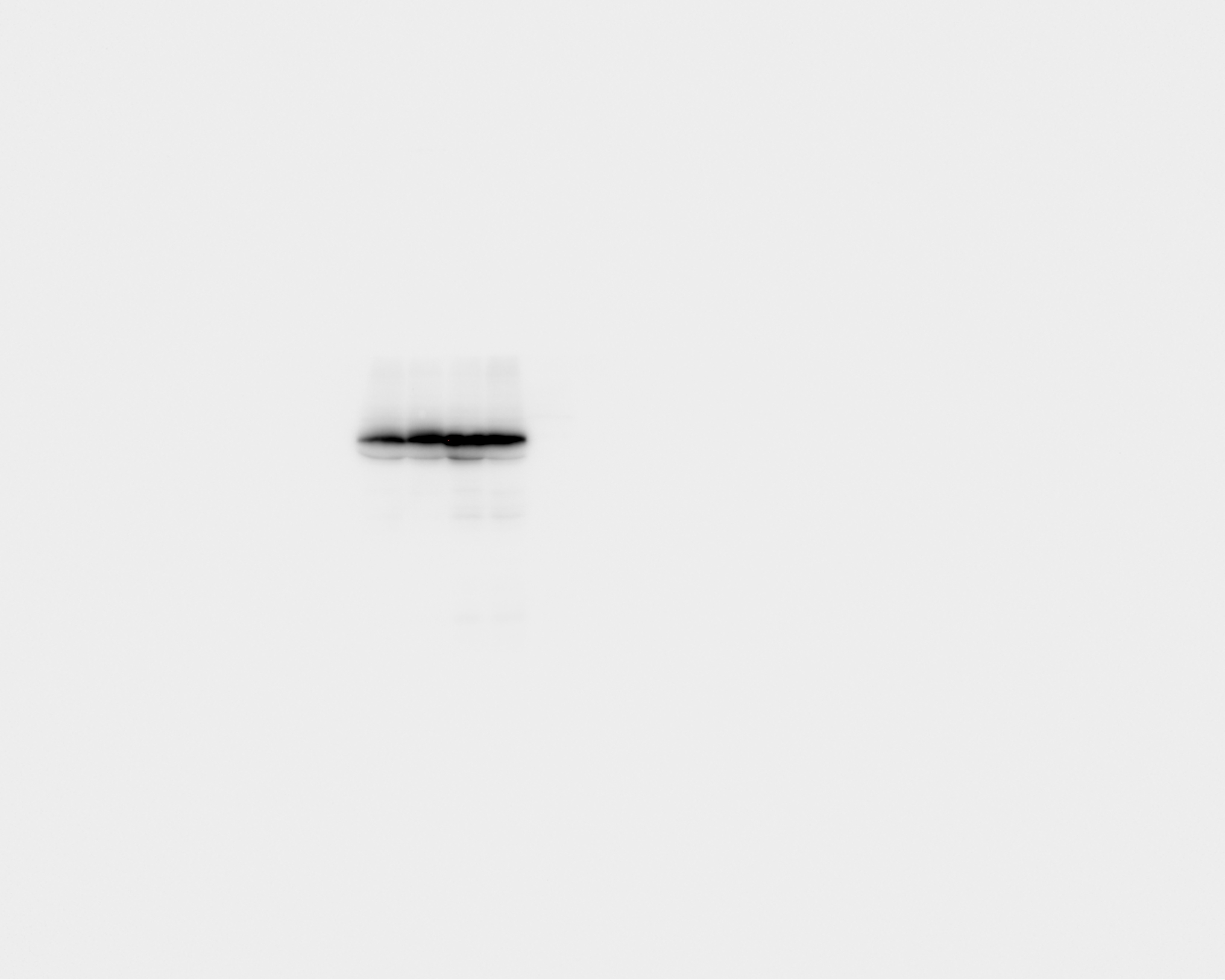

Supplement: Figure 4—figure supplement 2—source data 1. [file elife-92110-fig4-figsupp2-data1.zip › user 2023-07-19 18h41m41s(Chemiluminescence).jpg]

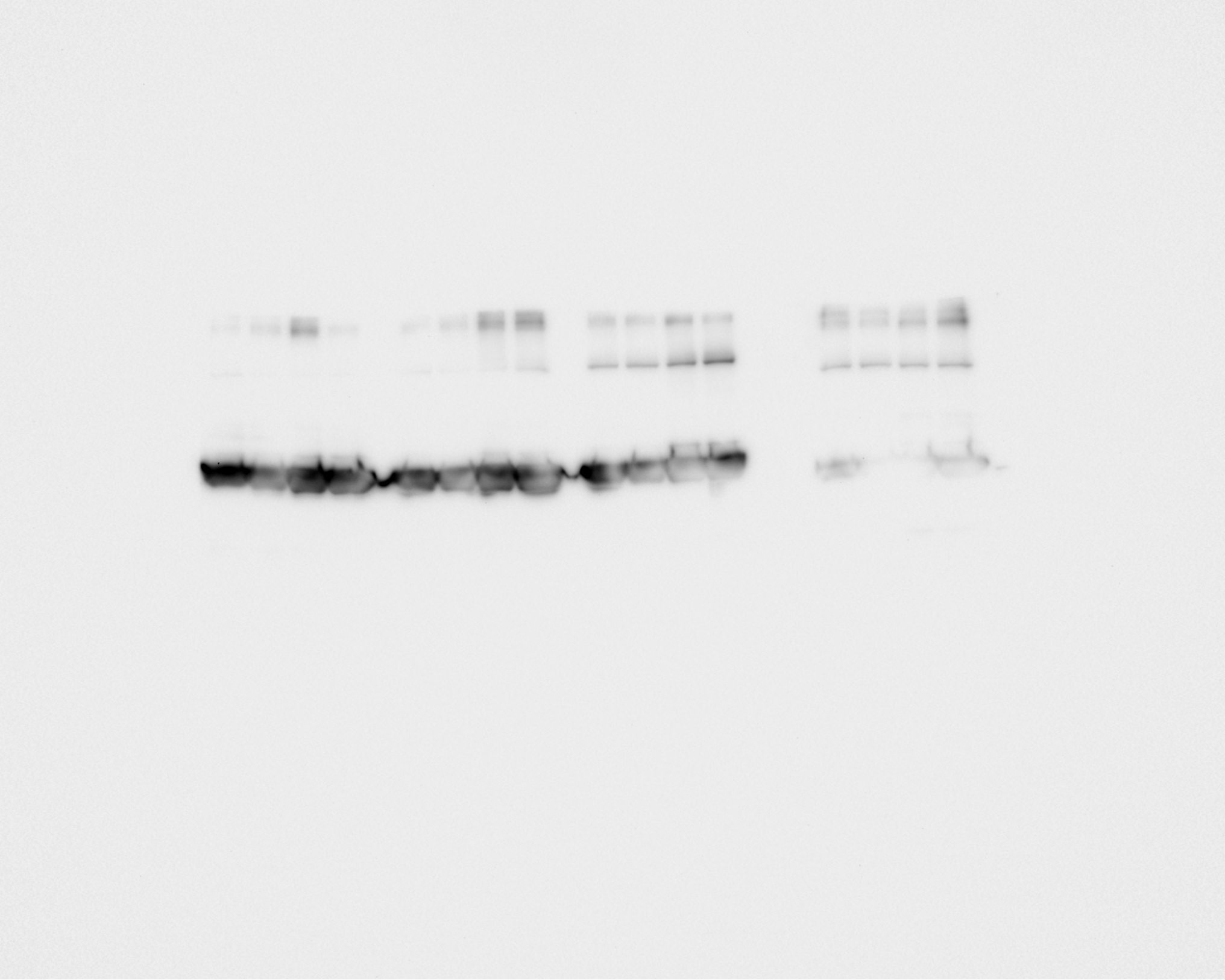

Supplement: Figure 4—figure supplement 2—source data 1. [file elife-92110-fig4-figsupp2-data1.zip › user 2023-07-19 18h51m07s(Chemiluminescence).jpg]

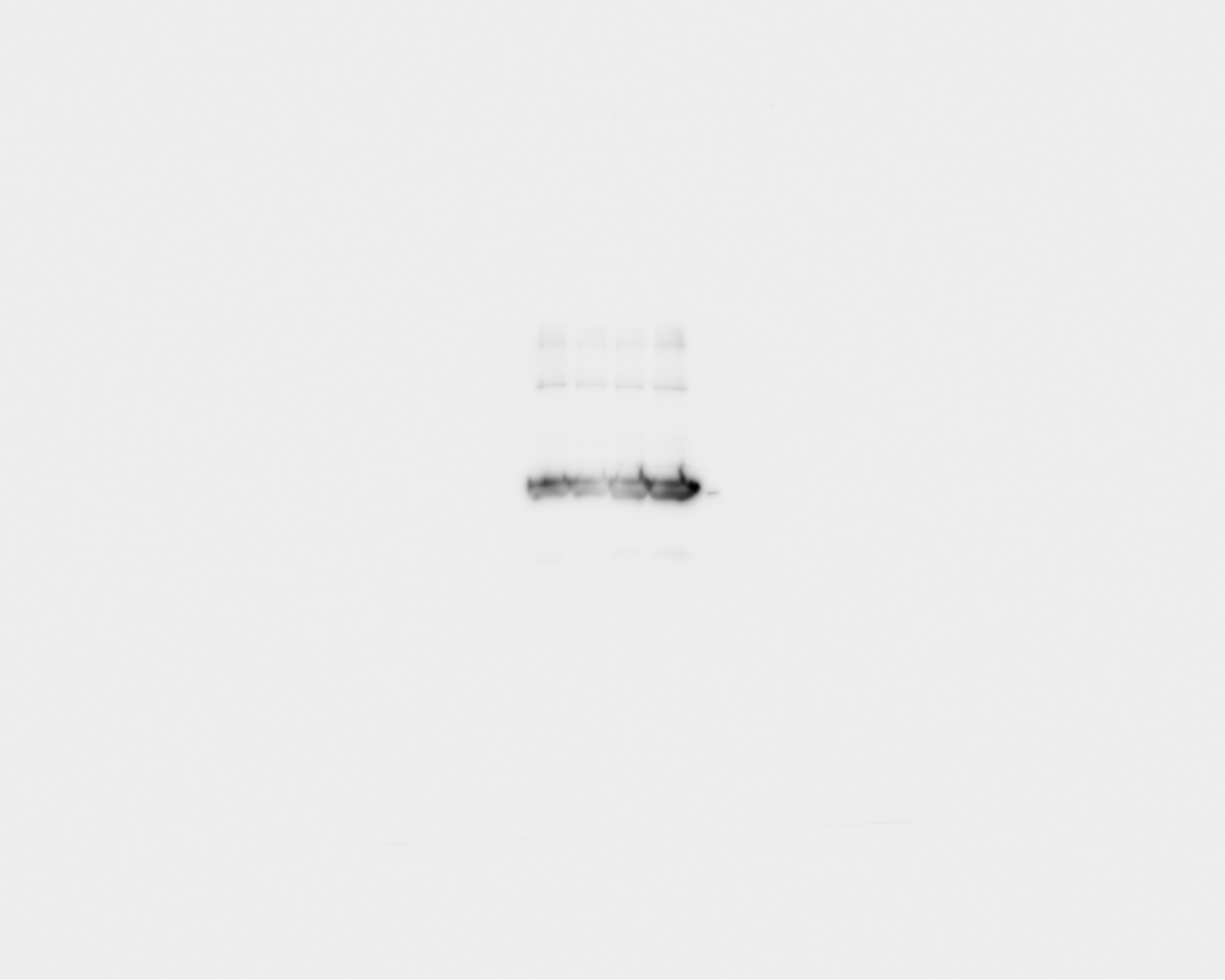

Supplement: Figure 4—figure supplement 2—source data 1. [file elife-92110-fig4-figsupp2-data1.zip › user 2023-07-19 19h04m56s(Chemiluminescence).jpg]

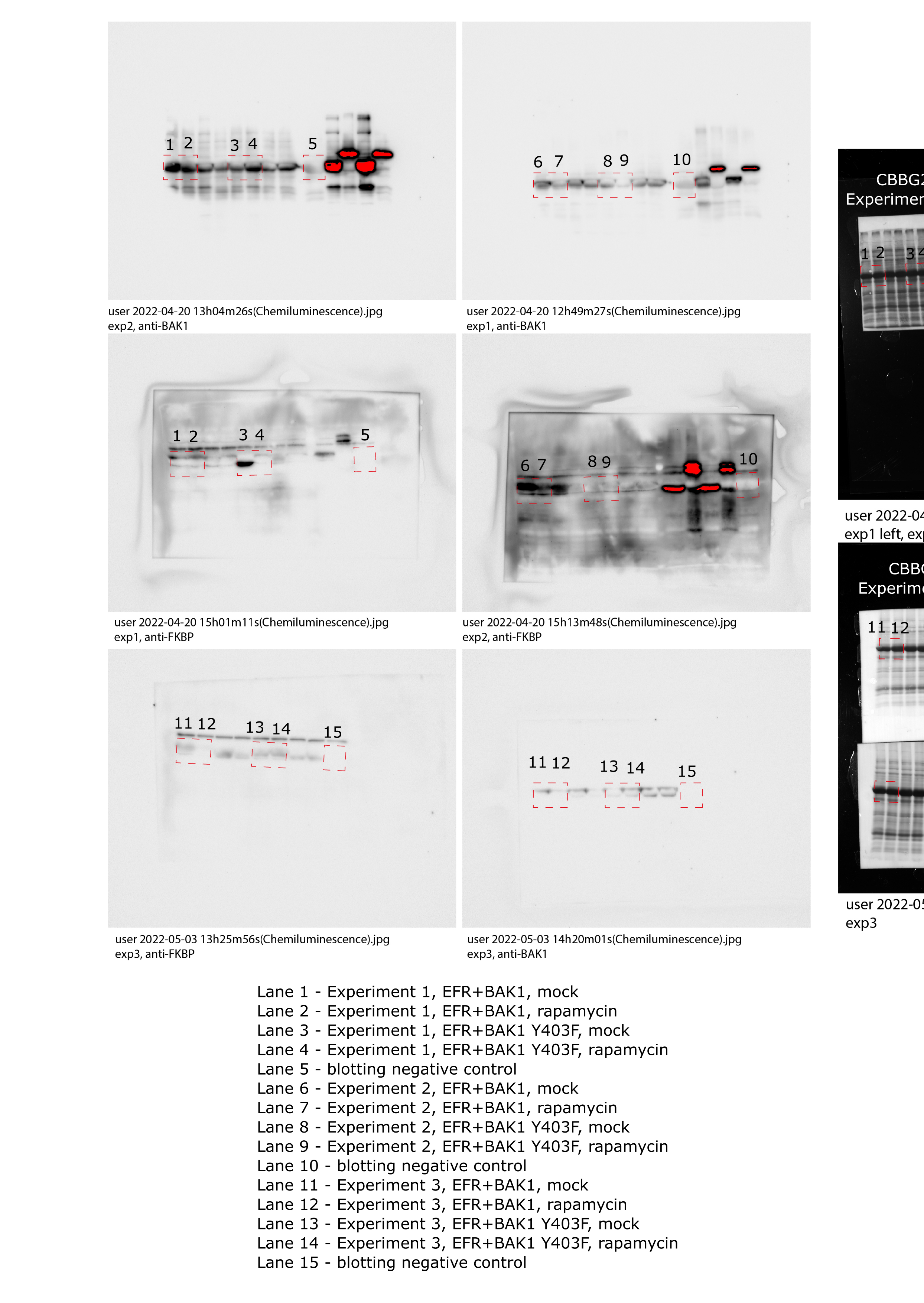

Supplement: Figure 4—figure supplement 3—source data 1. [file elife-92110-fig4-figsupp3-data1.zip › annotated.png]

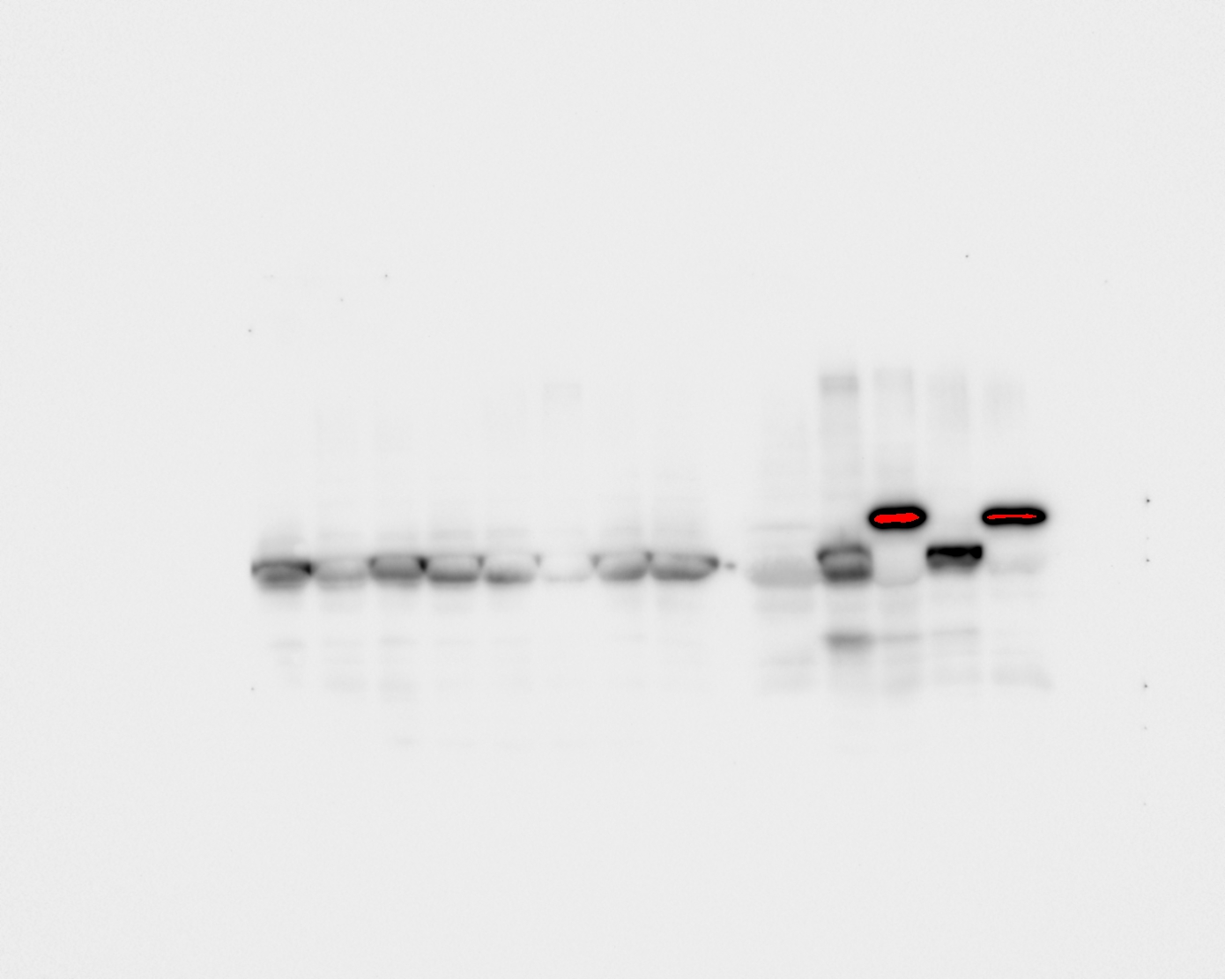

Supplement: Figure 4—figure supplement 3—source data 1. [file elife-92110-fig4-figsupp3-data1.zip › user 2022-04-20 12h49m27s(Chemiluminescence).jpg]

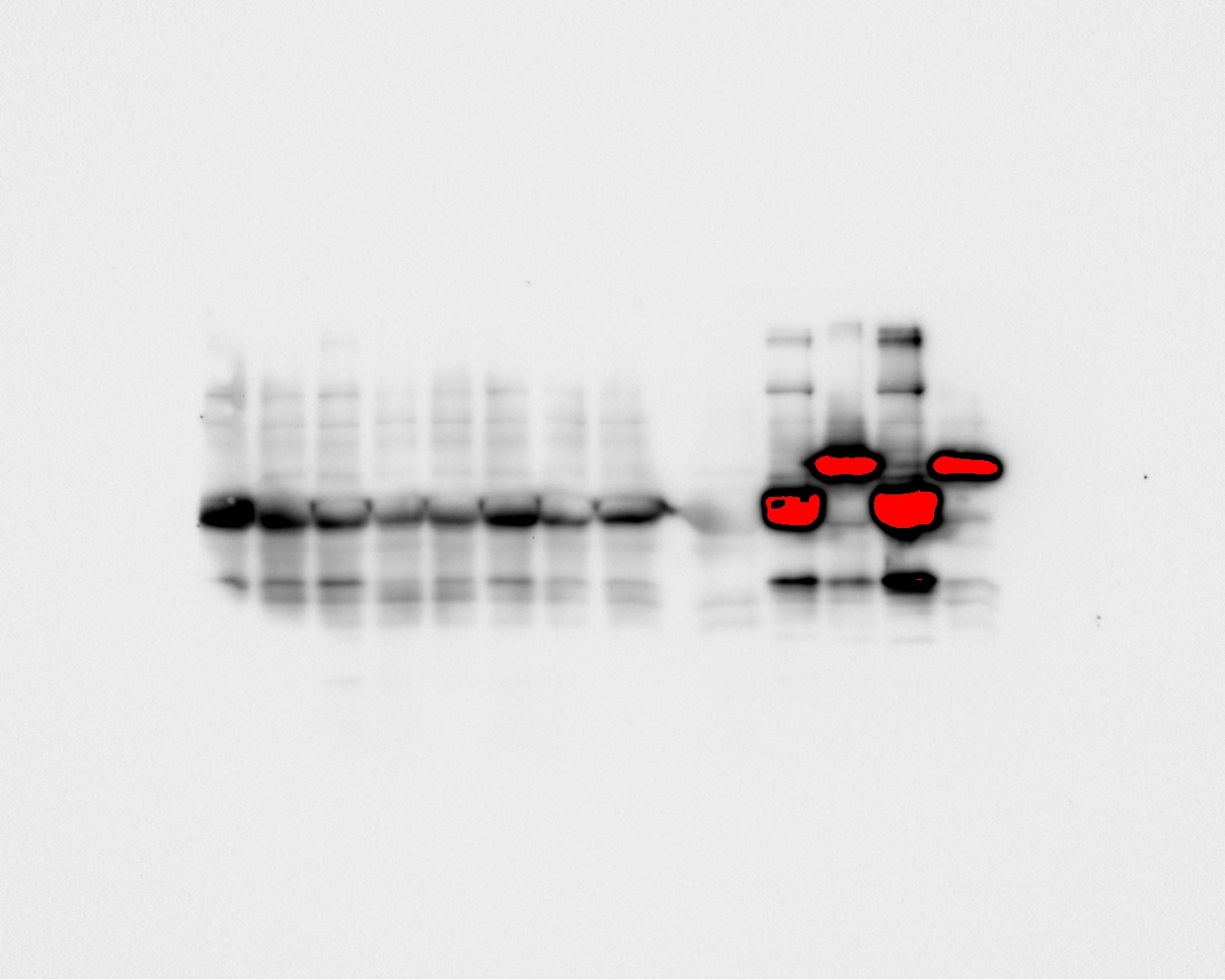

Supplement: Figure 4—figure supplement 3—source data 1. [file elife-92110-fig4-figsupp3-data1.zip › user 2022-04-20 13h04m26s(Chemiluminescence).jpg]

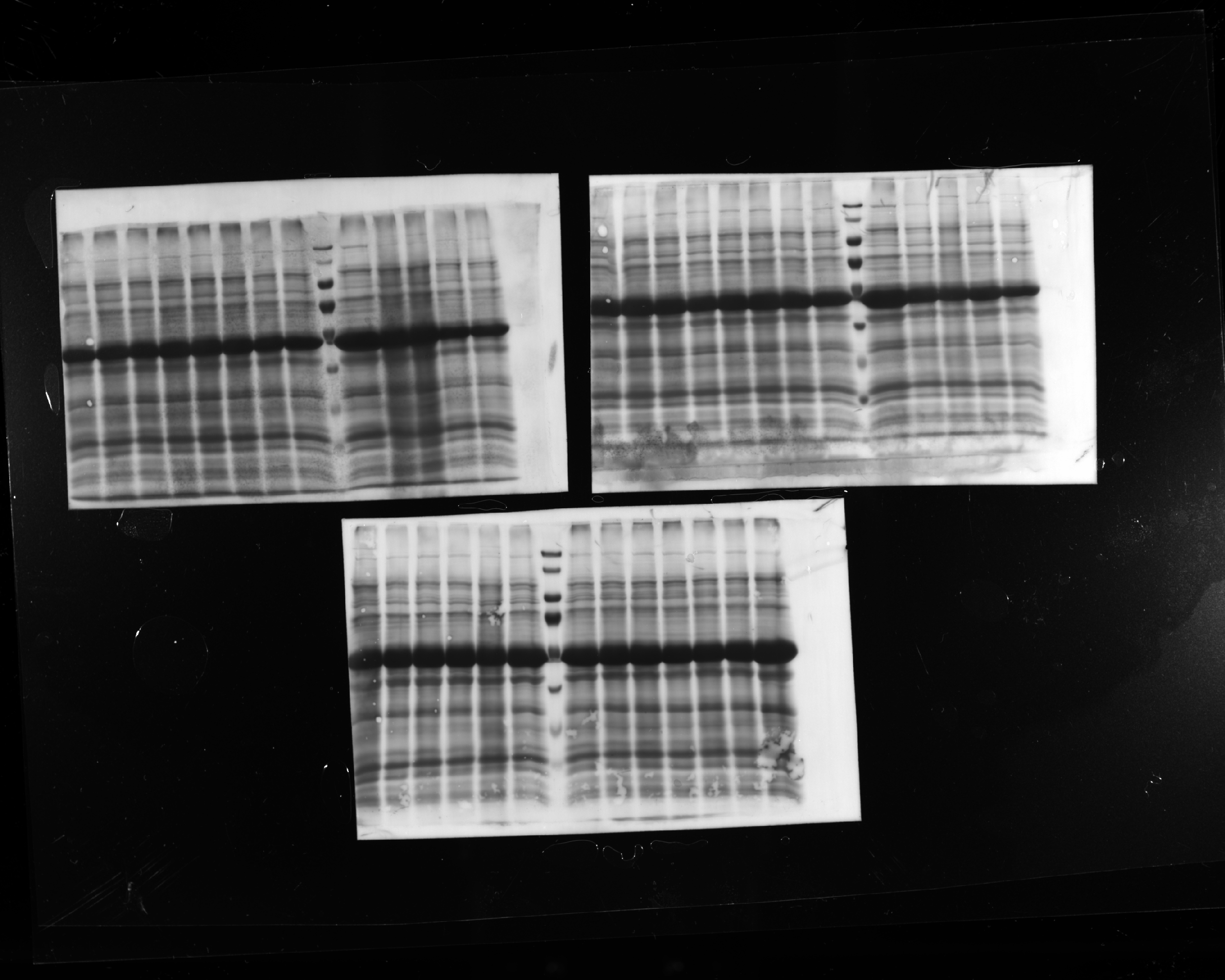

Supplement: Figure 4—figure supplement 3—source data 1. [file elife-92110-fig4-figsupp3-data1.zip › user 2022-04-20 14h45m07s(Colorimetric).jpg]

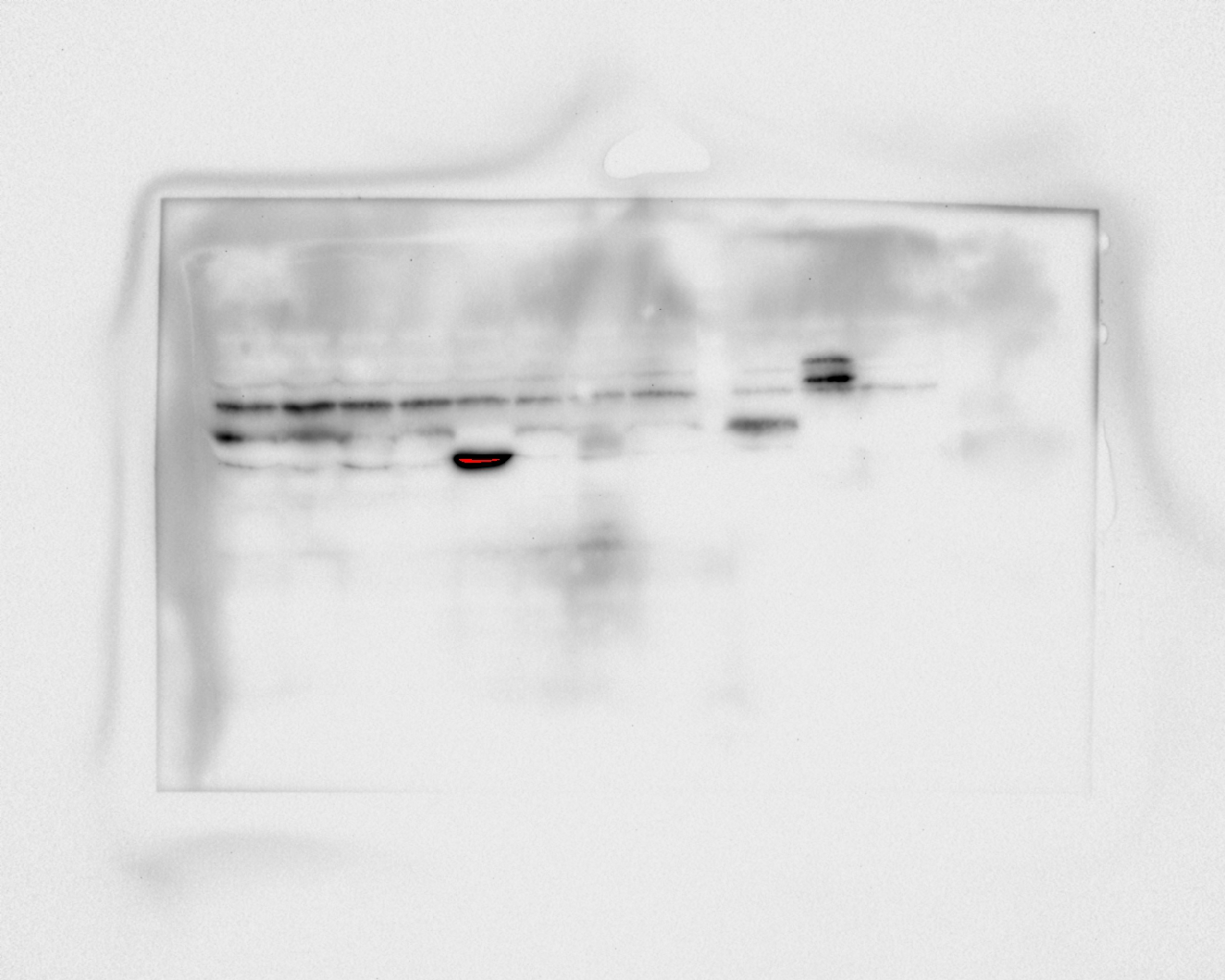

Supplement: Figure 4—figure supplement 3—source data 1. [file elife-92110-fig4-figsupp3-data1.zip › user 2022-04-20 15h01m11s(Chemiluminescence).jpg]

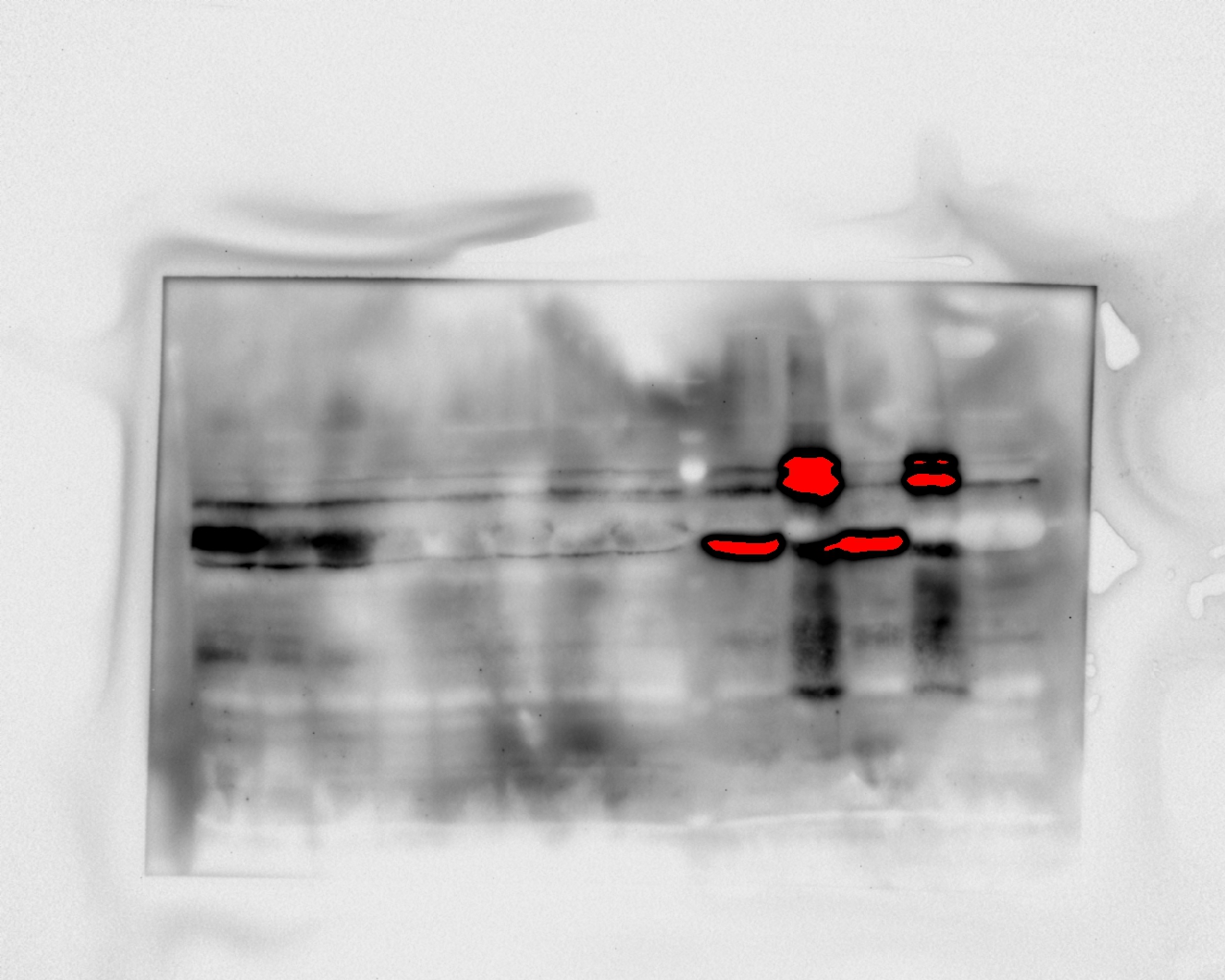

Supplement: Figure 4—figure supplement 3—source data 1. [file elife-92110-fig4-figsupp3-data1.zip › user 2022-04-20 15h13m48s(Chemiluminescence).jpg]

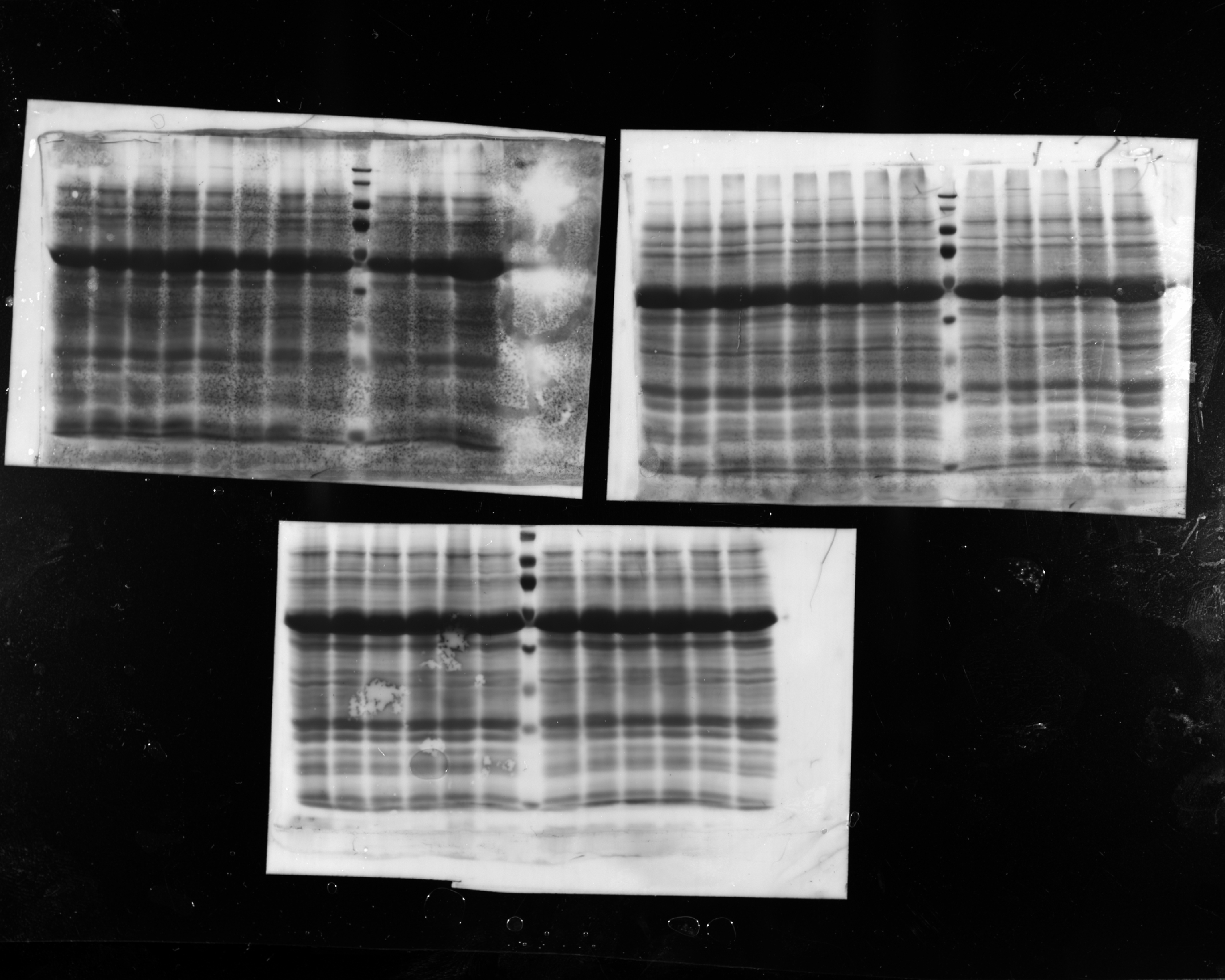

Supplement: Figure 4—figure supplement 3—source data 1. [file elife-92110-fig4-figsupp3-data1.zip › user 2022-04-21 05h44m34s(Colorimetric).jpg]

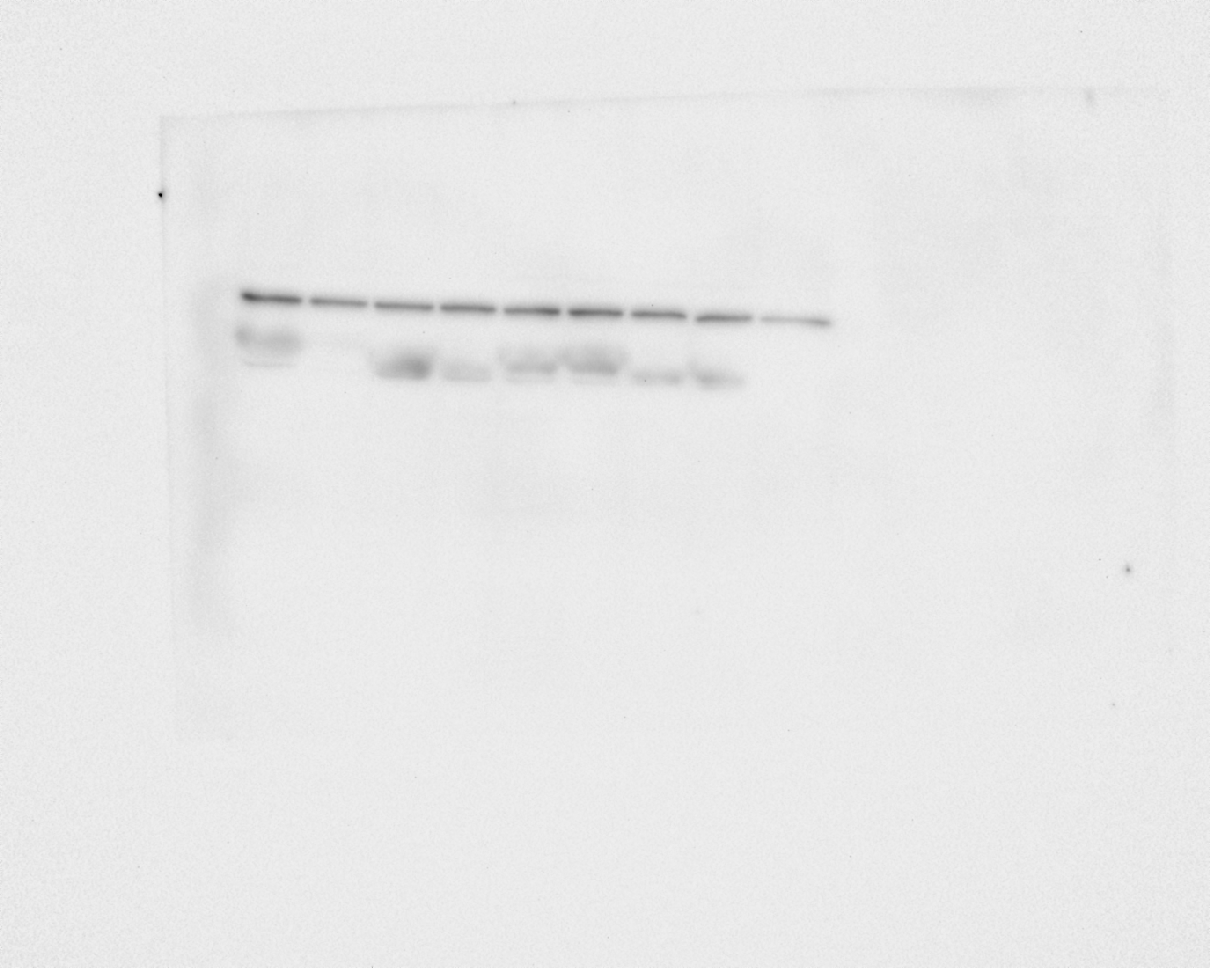

Supplement: Figure 4—figure supplement 3—source data 1. [file elife-92110-fig4-figsupp3-data1.zip › user 2022-05-03 13h25m56s(Chemiluminescence).jpg]

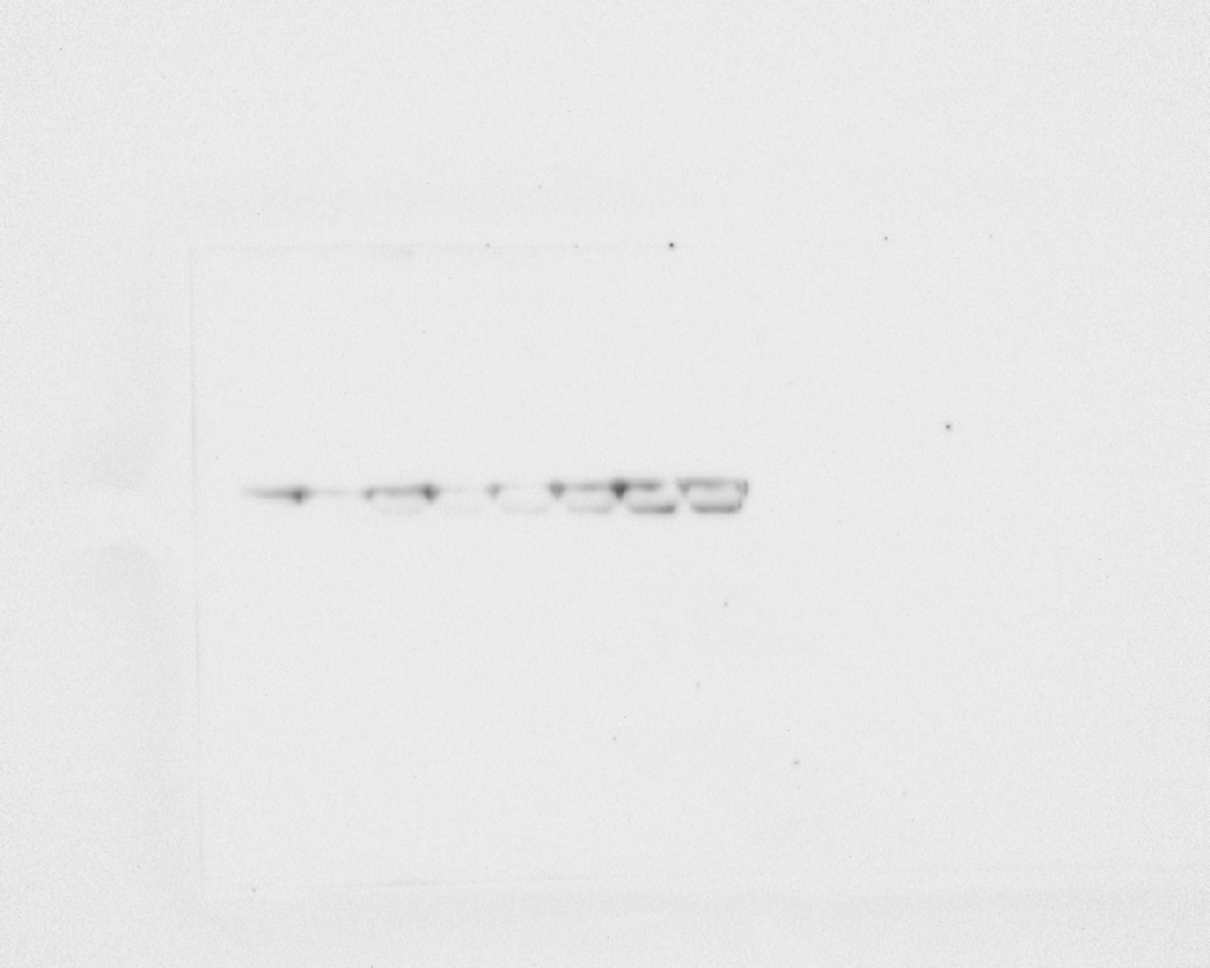

Supplement: Figure 4—figure supplement 3—source data 1. [file elife-92110-fig4-figsupp3-data1.zip › user 2022-05-03 14h20m01s(Chemiluminescence).jpg]

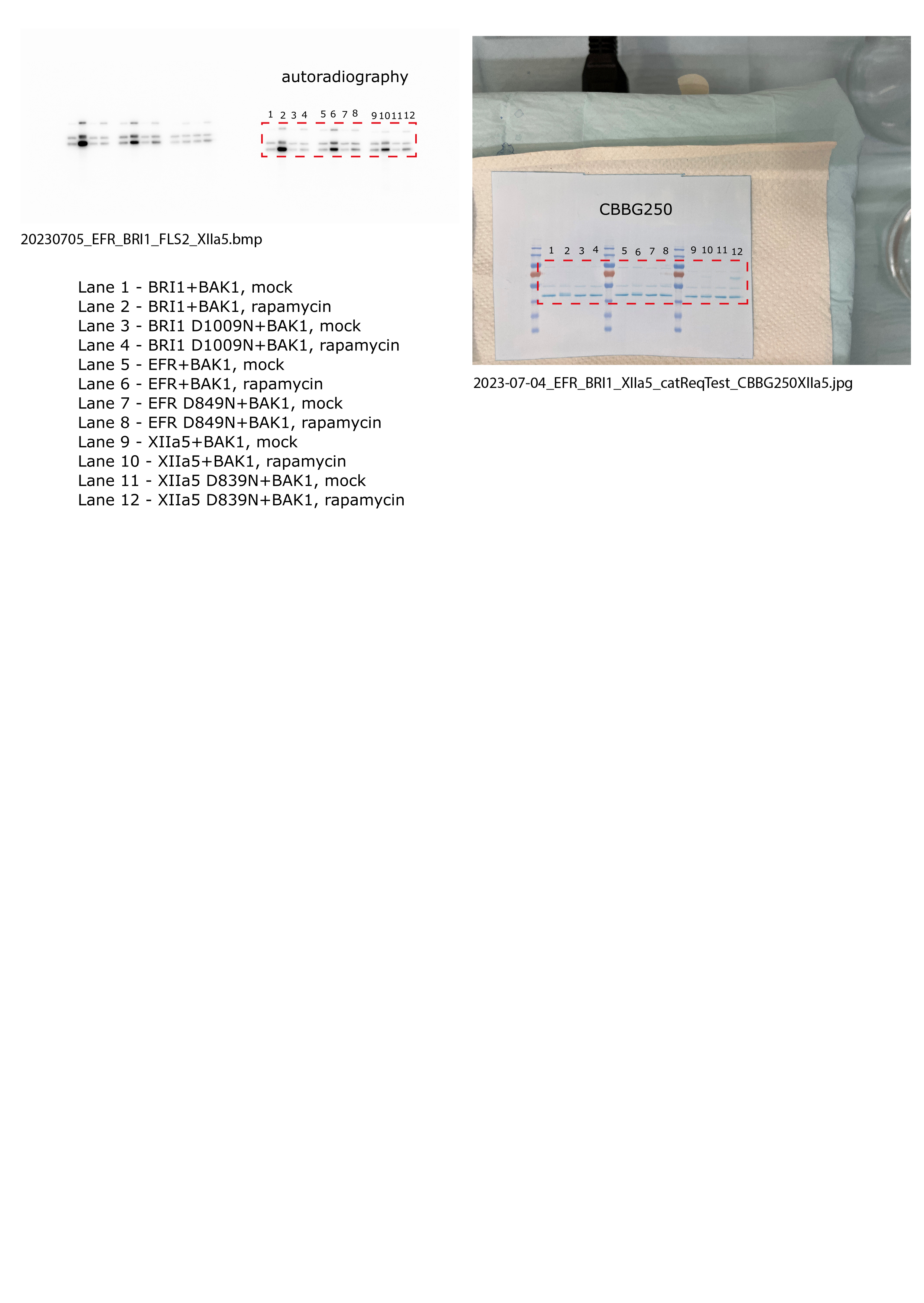

Supplement: Figure 5—figure supplement 1—source data 1. [file elife-92110-fig5-figsupp1-data1.zip › annotated.png]

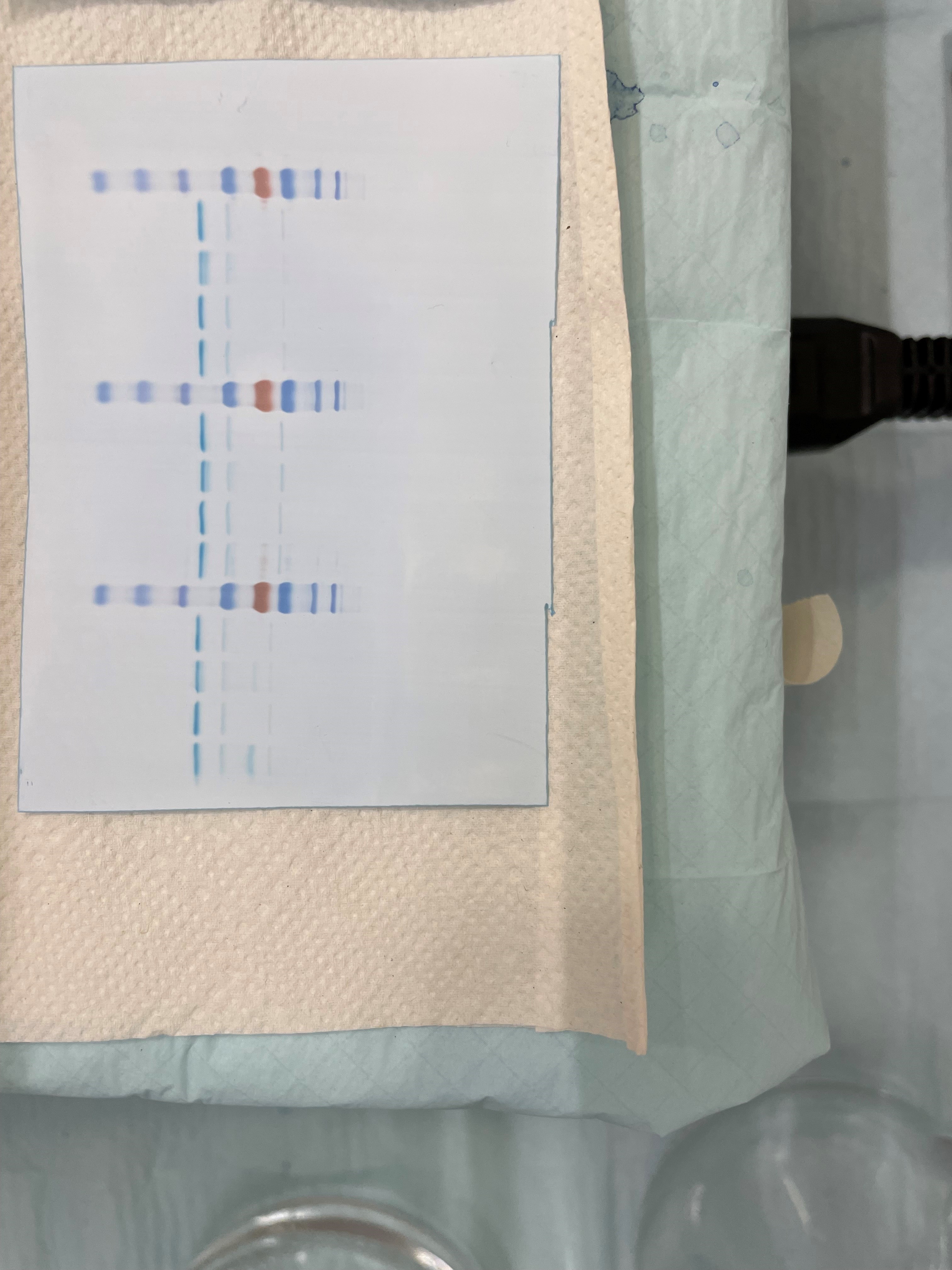

Supplement: Figure 5—figure supplement 1—source data 1. [file elife-92110-fig5-figsupp1-data1.zip › 2023-07-04_EFR_BRI1_XIIa5_catReqTest_CBBG250XIIa5.jpg]

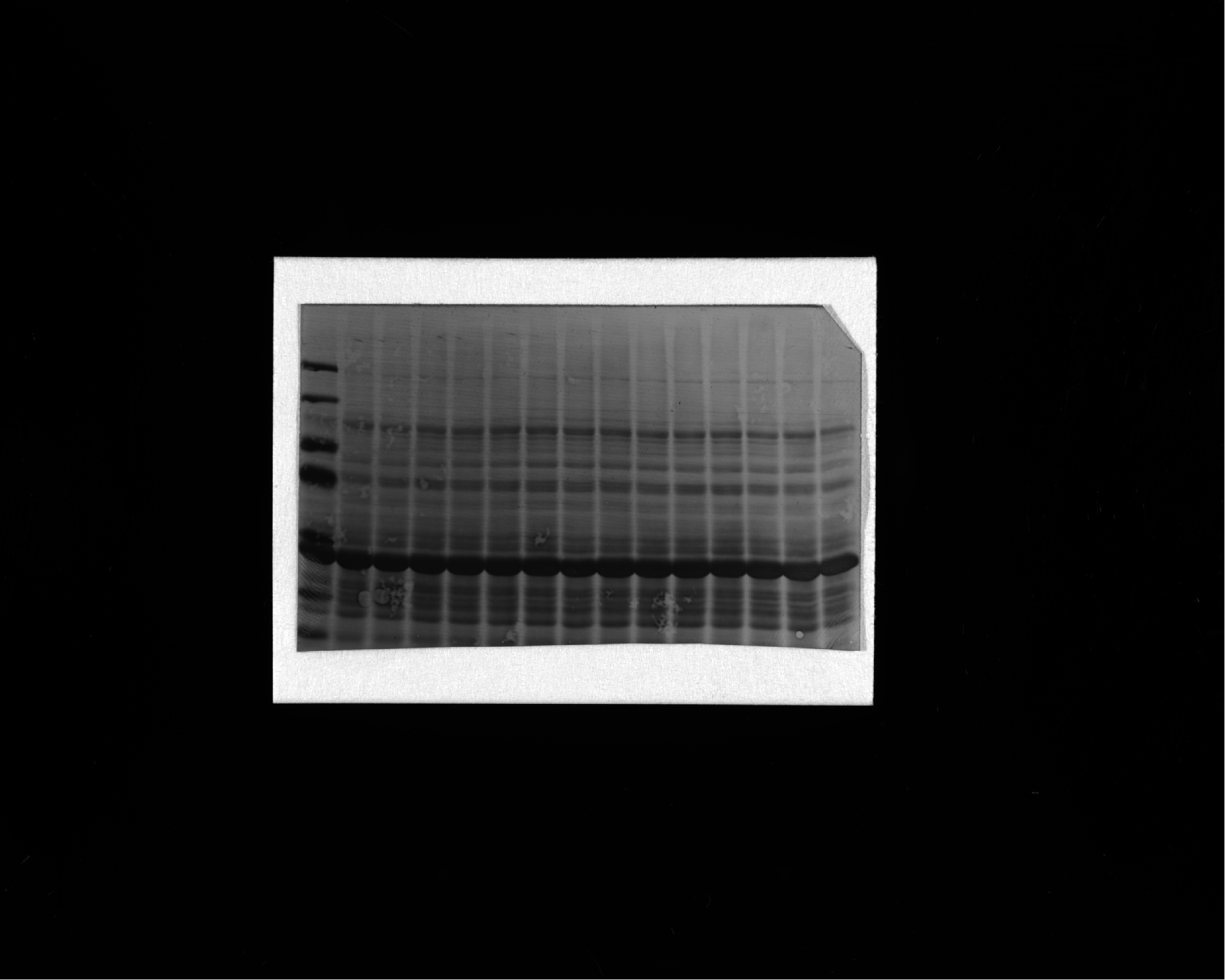

Supplement: Figure 5—figure supplement 2—source data 1. [file elife-92110-fig5-figsupp2-data1.zip › user 2023-08-11 11h19m27s.tif]

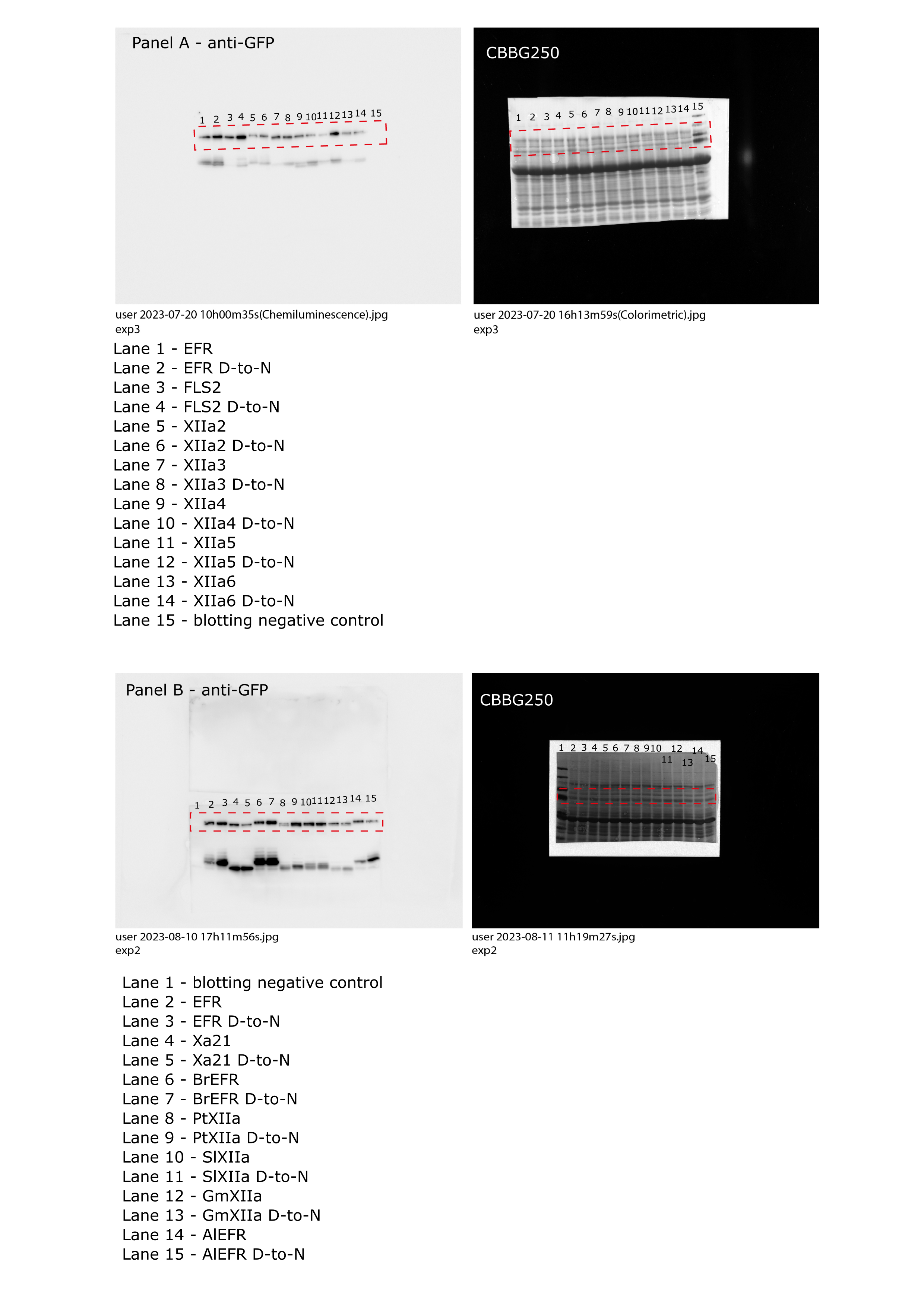

Supplement: Figure 5—figure supplement 2—source data 1. [file elife-92110-fig5-figsupp2-data1.zip › annotated.png]

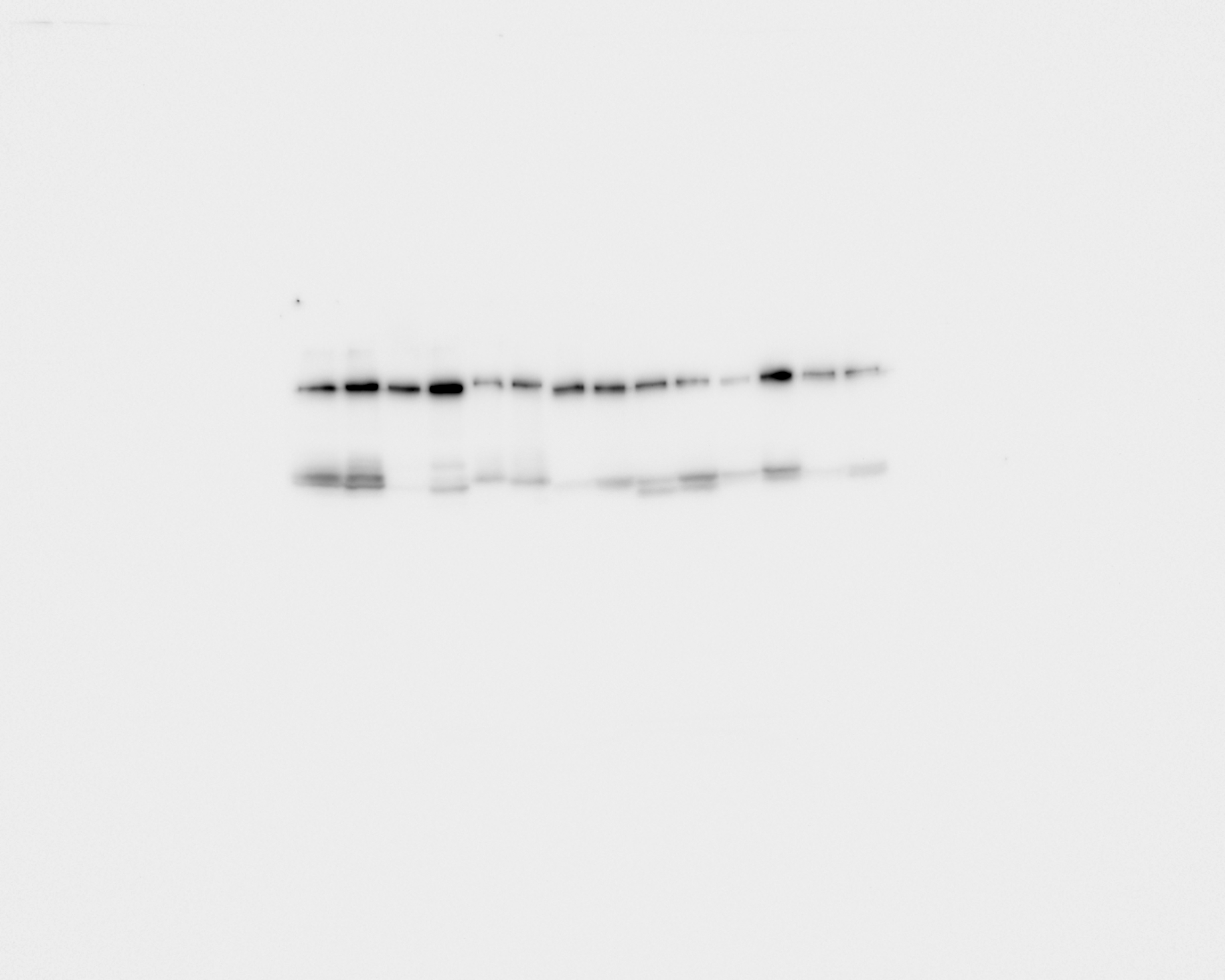

Supplement: Figure 5—figure supplement 2—source data 1. [file elife-92110-fig5-figsupp2-data1.zip › user 2023-07-20 10h00m35s(Chemiluminescence).jpg]

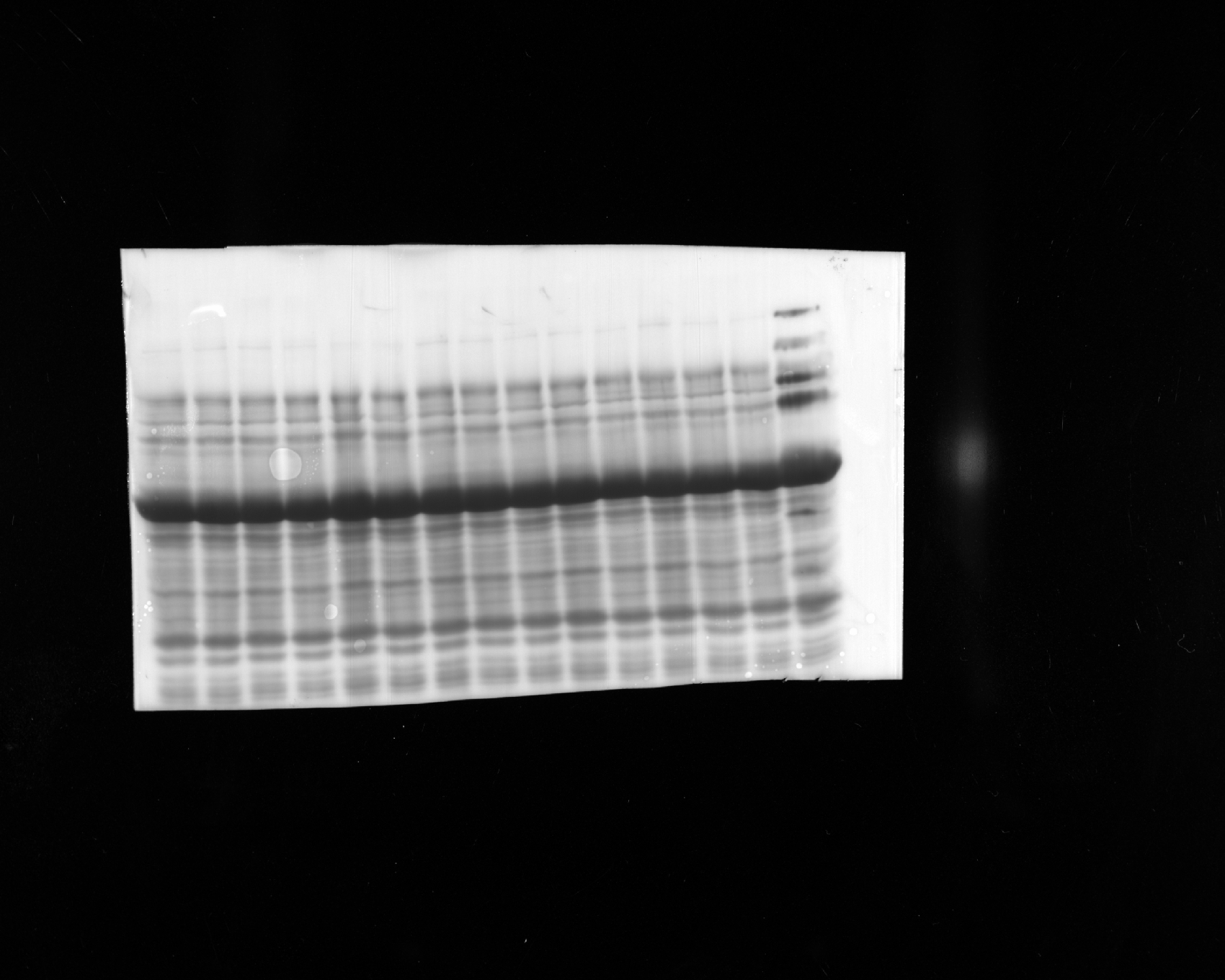

Supplement: Figure 5—figure supplement 2—source data 1. [file elife-92110-fig5-figsupp2-data1.zip › user 2023-07-20 16h13m59s(Colorimetric).jpg]

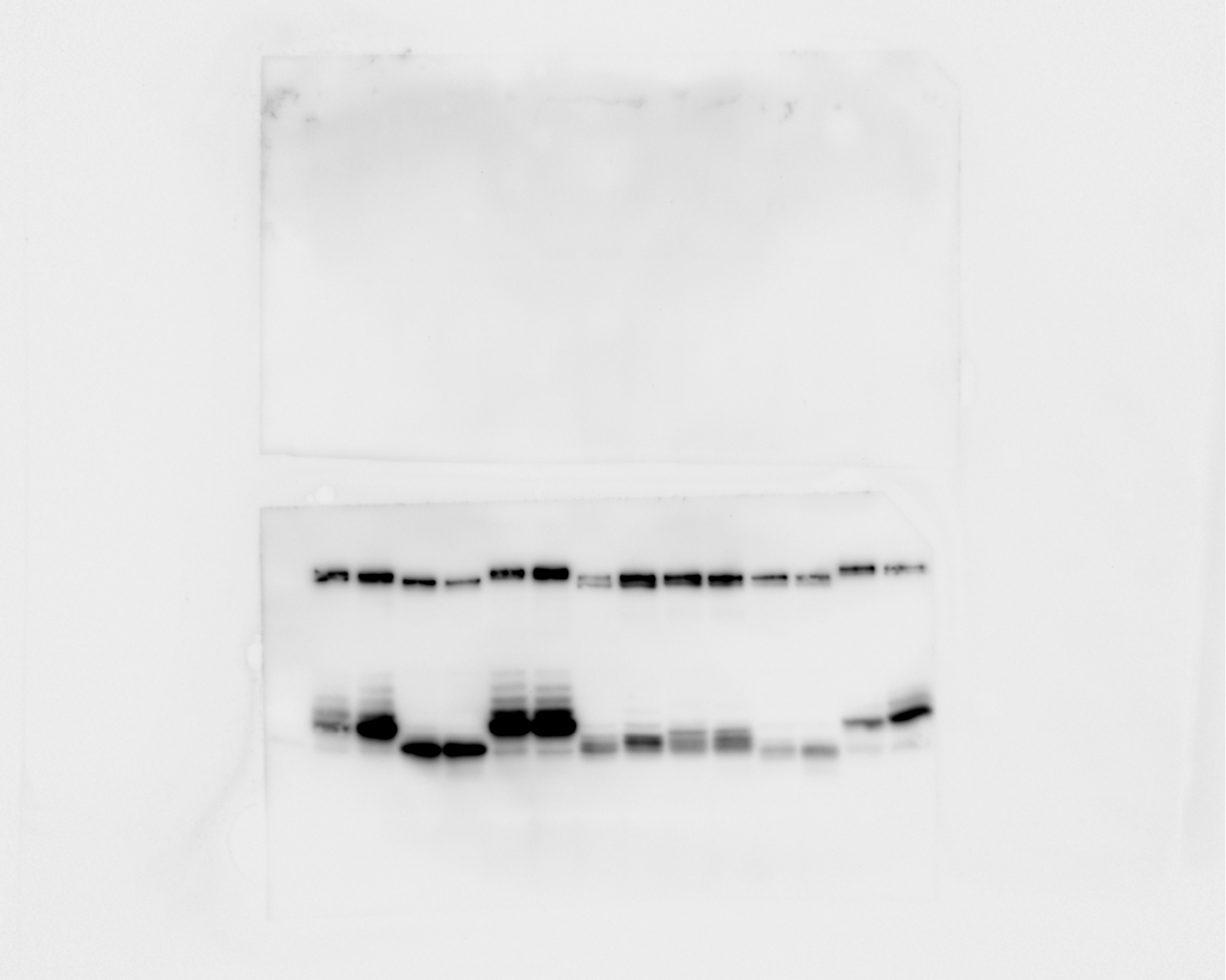

Supplement: Figure 5—figure supplement 2—source data 1. [file elife-92110-fig5-figsupp2-data1.zip › user 2023-08-10 17h11m56s.jpg]
